# New Insights on *Streptococcus dysgalactiae* subsp. *dysgalactiae* Isolates

**DOI:** 10.3389/fmicb.2021.686413

**Published:** 2021-07-15

**Authors:** Cinthia Alves-Barroco, João Caço, Catarina Roma-Rodrigues, Alexandra R. Fernandes, Ricardo Bexiga, Manuela Oliveira, Lélia Chambel, Rogério Tenreiro, Rosario Mato, Ilda Santos-Sanches

**Affiliations:** ^1^UCIBIO, Departamento de Ciências da Vida, NOVA School of Science and Technology/FCT NOVA, Universidade NOVA de Lisboa, Caparica, Portugal; ^2^Centro de Investigação Interdisciplinar em Sanidade Animal, Faculdade de Medicina Veterinária, Universidade de Lisboa, Lisbon, Portugal; ^3^Biosystems and Integrative Sciences Institute, Faculdade de Ciências, Universidade de Lisboa, Edifício TecLabs, Lisbon, Portugal

**Keywords:** *Streptococcus dysgalactiae* subsp. *dysgalactiae*, CRISPR typing, phylogenetic relationships, virulence genes, phage genes

## Abstract

*Streptococcus dysgalactiae* subsp. *dysgalactiae* (SDSD) has been considered a strict animal pathogen. Nevertheless, the recent reports of human infections suggest a niche expansion for this subspecies, which may be a consequence of the virulence gene acquisition that increases its pathogenicity. Previous studies reported the presence of virulence genes of *Streptococcus pyogenes* phages among bovine SDSD (collected in 2002–2003); however, the identity of these mobile genetic elements remains to be clarified. Thus, this study aimed to characterize the SDSD isolates collected in 2011–2013 and compare them with SDSD isolates collected in 2002–2003 and pyogenic streptococcus genomes available at the National Center for Biotechnology Information (NCBI) database, including human SDSD and *S. dysgalactiae* subsp. *equisimilis* (SDSE) strains to track temporal shifts on bovine SDSD genotypes. The very close genetic relationships between humans SDSD and SDSE were evident from the analysis of housekeeping genes, while bovine SDSD isolates seem more divergent. The results showed that all bovine SDSD harbor Clustered Regularly Interspaced Short Palindromic Repeats (CRISPR)/Cas IIA system. The widespread presence of this system among bovine SDSD isolates, high conservation of repeat sequences, and the polymorphism observed in spacer can be considered indicators of the system activity. Overall, comparative analysis shows that bovine SDSD isolates carry *speK*, *speC*, *speL*, *speM*, *spd1*, and *sdn* virulence genes of *S. pyogenes* prophages. Our data suggest that these genes are maintained over time and seem to be exclusively a property of bovine SDSD strains. Although the bovine SDSD genomes characterized in the present study were not sequenced, the data set, including the high homology of superantigens (SAgs) genes between bovine SDSD and *S. pyogenes* strains, may indicate that events of horizontal genetic transfer occurred before habitat separation. All bovine SDSD isolates were negative for genes of operon encoding streptolysin S, except for *sagA* gene, while the presence of this operon was detected in all SDSE and human SDSD strains. The data set of this study suggests that the separation between the subspecies “*dysgalactiae*” and “*equisimilis*” should be reconsidered. However, a study including the most comprehensive collection of strains from different environments would be required for definitive conclusions regarding the two taxa.

## Introduction

*Streptococcus dysgalactiae* (SD) includes two subspecies: *S. dysgalactiae* subsp. *dysgalactiae* (SDSD) and *S. dysgalactiae* subsp. *equisimilis* (SDSE) (Jensen and Kilian, 2012). While SDSE has been recognized as an increasingly important human pathogen, SDSD has been considered a strictly animal pathogen and is commonly associated with bovine mastitis (Zadoks et al., 2011; Rato et al., 2013). In the last years, although rare, human diseases by this subspecies have been reported (Koh et al., 2009; Park et al., 2012; Jordal et al., 2015; Chennapragada et al., 2018), and it is urgent to understand the role of this subspecies in human pathogenesis. Recently, we have reported bovine SDSD isolates’ ability to adhere to and internalize different human cell lines, including keratinocytes (Roma-Rodrigues et al., 2016; Alves-Barroco et al., 2018).

SDSD and SDSE, together with *Streptococcus agalactiae*, *Streptococcus canis*, and *Streptococcus pyogenes*, are the major pathogens of the pyogenic group. Previous studies reveal that these species are closely related. Phylogenetic analysis involving the *Streptococcus* core genome within the pyogenic clade displayed overall consensus. Though the issue is not entirely clear-cut, the most likely scenario is that the species tree has *S. dysgalactiae* and *S. pyogenes* as sister species and, together with *S. canis*, establishes a very closely related branch. These data suggest that they have recently descended from a common ancestor (Suzuki et al., 2011; Jensen and Kilian, 2012; Lefébure et al., 2012). The divergence among species has been mainly associated with mobile genetic elements (MGEs) (Lefébure et al., 2012). Indeed, about a quarter of streptococci’s genome consists of exogenous and mobile DNA, e.g., transposons, prophage, and plasmid. Thus, horizontal gene transfer (HGT) is considered the primary mechanism for generating diversity in this genus (Baiano and Barnes, 2009; Haenni et al., 2010; De la Casa-Esperón, 2012; Richards et al., 2012; Wong and Yuen, 2012; Rohde and Cleary, 2016). Overall, the MGEs are self-transmissible elements that are omnipresent in bacteria, harboring genes responsible for their maintenance, regulation, and mobility and genes encoding antimicrobial resistance, as well as other determinants, such as genes that encode virulence factors (Grohmann et al., 2003; McNeilly and McMillan, 2014).

Thus, many MGEs serve as shuttles for genes that are beneficial to bacteria during their proliferation in a host environment. Although several mechanisms can mediate genetic transfer in pyogenic streptococci, the transduction might be particularly relevant since bacteriophages have been detected in a considerable proportion (McShan and Nguyen, 2016). In *S. pyogenes* and SDSE, more than 20% of the accessory genomes consist of prophage DNA, highlighting the critical role of transduction in the evolution of these bacteria (Lier et al., 2015; Yamada et al., 2019).

The presence of phage-carrying *S. pyogenes* virulence genes among SDSD isolates from bovine mastitis was previously reported, namely, the streptococcal pyrogenic exotoxin genes (*speK*, *speC*, *speL*, and *speM*), the phage DNase (*spd*1), and determinants of antibiotic resistance located on MGEs (Rato et al., 2011; Abdelsalam et al., 2015). It was hypothesized that these virulence genes might contribute to the increased pathogenic potential; thus, SDSD should not be ignored as a potential human pathogen.

Despite the benefits of HGT, due to the vast diversity of the bacteriophages in the environment, the bacteria are under constant phage attack. To survive, the bacteria have devised various strategies that involve innate and adaptive immunity and interfere with the infection process (Barrangou and Marraffini, 2014). Among all of these strategies, the Clustered Regularly Interspaced Short Palindromic Repeats (CRISPR)/Cas system is the one that provides the bacteria with the ability to “memorize” a piece of the invaders’ genetic material (Bhaya et al., 2011; Bondy-Denomy et al., 2013; Rath et al., 2015; Makarova et al., 2015; Makarova et al., 2018) by acquiring foreign nucleic acid fragments called spacers. Together with host Cas proteins, the spacers behave as an adaptive immune system in bacteria and archaea. Besides, the sequential order of the spacers in the array provides chronological information about the bacteria’s exposure to foreign nucleic acids (Lier et al., 2015), providing insights into host–phage interactions within a specific niche. These data show indicators of the evolution of the bacteria that carry them (Lier et al., 2015).

To date, studies on the presence of *S. pyogenes* phages among SDSD isolates are scarce. For example, it is not yet clear whether these phages (and other MGEs) contribute to more pathogenic SDSD phenotypes. The CRISPR/Cas system in the SDSD and its impact on HGT are examples of how little is known, so far, about this microorganism.

In the present study, SDSD isolates collected in two different periods (2002–2003, partially studied, and 2011–2013) were characterized and compared to obtain new information about molecular virulence profiles, antibiotypes, and population structure. We also investigated the CRISPR/Cas systems in SDSD strains to gain insights into defense systems and their impact on HGT. Additionally, pyogenic streptococcus genomes available at National Center for Biotechnology Information (NCBI) database, including human SDSD and SDSE strains, were included in this study to characterize CRISPR/Cas system, prophage regions, giving further insights on horizontal acquisitions of genetic elements particularly associated with resistance traits or virulence to track temporal shifts of SDSD genotypes.

## Materials and Methods

### Bacterial Isolates

Out of a total of 194 Gram-positive cocci, 37 SDSD were isolated and included in this study (collection II). The isolates were collected from 2011 to 2013, from 250 animals with mastitis of 12 dairy farms located in Portugal. The presumptive identification of the SDSD isolates was performed by traditional phenotypic tests such as colony morphology, type of hemolysis, Lancefield serologic groups using the SLIDEX Strepto Plus (Biomérieux, Marcy-l’Étoile, France), and absence of catalase.

For a comparative analysis, 18 strains of bovine SDSD (collection I), partially characterized (Rato et al., 2010, 2011, 2013) and collected between 2002 and 2003 from 248 animals with mastitis on eight farms located in Portugal, were included in the study.

### Genomic DNA Extraction

Genomic DNA was extracted according to Alves-Barroco et al. (2019).

### Identification by 16S rRNA Sequence

The identification of the SDSD isolates was confirmed by analysis of 16S rRNA gene sequencing. Amplification and sequencing were performed with the same primers (Takahashi et al., 1997). The sequencing of PCR products was carried out by STAB-Vida (Lisbon, Portugal). The sequences were analyzed and aligned using CLC Main Workbench 20.1 alignment program editor (QIAGEN, Venlo, Netherlands) and then compared with sequences from the NCBI database using the Basic Local Alignment Search Tool (BLAST)^1^. Subsequently, the nucleotide sequences were subjected to phylogenetic analysis using MEGA X (Kumar et al., 2018).

### Pulsed-Field Gel Electrophoresis

The agarose plugs containing the genomic DNA from streptococcal strains were prepared as previously described (Rato et al., 2008). All pulsed-field gel electrophoresis (PFGE) patterns were analyzed visually and by computer-assisted cluster analysis using BioNumerics v. 6.6 (Applied Maths, Sint-Martens-Latem, Belgium). Levels of similarity were calculated using Dice coefficient, and unweighted pair group method with arithmetic mean (UPGMA) was used for clustering to produce band-based dendrograms (1.5% and no optimization). Groups of patterns with similarity of 100% were considered indistinguishable and were assigned to the same subtype of PFGE type.

### Phylogenetic Analysis

For the phylogenetic analysis of bovine SDSD isolates, the Multilocus Sequence Analysis (MLSA) scheme was performed according to the method previously described for SDSE based on sequences of seven housekeeping genes (McMillan et al., 2010). Seven housekeeping genes, namely, glucose kinase *gki*, glutamine transport protein *gtr*, glutamate racemase *murI*, DNA mismatch repair protein *mutS*, transketolase *recP*, xanthine phosphoribosyl transferase *xpt*, and acetoacetyl-coathioloase *atoB*, were amplified; and all amplified DNA fragments were sequenced. The sequences were edited, aligned, and subjected to phylogenetic analysis using CLC Bio Main Workbench 20.1 alignment program editor (QIAGEN, Venlo, Netherlands). Housekeeping gene sequences were trimmed, concatenated, and subjected to phylogenetic analysis. For concatenated housekeeping alleles, phylogenetic relationships were inferred using the minimum evolution method, and the distances were computed using the maximum composite likelihood method.

For the phylogenetic analysis, bovine SDSD isolates representatives of collection I and II (a total of 15 strains) were selected based on their different resistance, virulence, and PFGE profiles. Additionally, the sequences of SDSD strains (*n* = 12), SDSE strains (*n* = 15), *S. pyogenes* strains (*n* = 11), and *S. canis* strains (*n* = 3) (Supplementary Table 1 in Supplementary File 1), available in the NCBI Nucleotide database, were included in this study for comparison.

### Virulence Gene Screening

The following genetic determinants of *S. pyogenes* (i.e., *speA*, *speB*, *speC*, *speF*, *speG*, *speH*, *speK*, *speL*, *speM*, *spegg*, *smeZ*, *spd1*, and *sdn*) were screened in bovine SDSD isolates by PCR. Additionally, we investigated the presence of operon encoding streptolysin S (SLS), namely, *sagA*, *sagB*, *sagE*, and *sagI* genes. For each 25 μl PCR mixture were added 100 ng of bacterial DNA, 1 × reaction buffer for NZYTaq DNA polymerase, 2.5 mM of MgCl_2_, 0.4 mM of dNTPs NZYMix, 1 U of NZYTaq DNA polymerase (NZYTech, Lisbon, Portugal), and 1 μM of each primer (Thermo Fisher Scientific, Waltham, MA, United States). Gene expression assays by reverse-transcriptase PCR (RT-PCR) were performed according to Alves-Barroco et al. (2019). Negative results were confirmed in at least three independent assays. VSD13 and VSD7 were used as positive control. Primer sequences and amplicon expected size PCR conditions for amplifying all the genetic determinants are listed in Supplementary Table 2 (Supplementary File 2).

Screening of virulence genes and comparative analysis *in silico* among SDSD (from bovine, human, and fish strains), SDSE, *S. canis*, and *S. pyogenes* sequences (see Supplementary Table 1 in Supplementary File 1) were performed using the CLC Bio Main Workbench. Alignment of *speC*, *speK*, *speL*, *speM*, and *spd1* sequence of bovine SDSD against the GenBank database using BLAST was performed to trace homolog genes among streptococcal species.

### Antimicrobial Resistance Patterns

All SDSD strains were tested for antimicrobial resistance by disk diffusion method (Oxoid Ltd., Basingstoke, United Kingdom), and the determination of minimum inhibitory concentration (MIC) follows the guidelines from the Clinical and Laboratory Standards Institute (CLSI) for antimicrobial susceptibility tests for bacteria isolated from animals (CLSI M31-A3, 2008). The following antimicrobials were selected for testing, based on several criteria: (a) licensing for mastitis treatment in cattle—amoxicillin–clavulanic acid 30 μg (AMC), gentamicin 10 μg (CN), penicillin 10 units (P), pirlimycin 2 μg (PRL), and streptomycin 10 μg (S); and (b) use in human medicine—chloramphenicol 30 μg (CHL), erythromycin 15 μg (ERY), tetracycline 30 μg (TET), and vancomycin 30 μg (VA). The results were interpreted according to the CLSI guidelines (CLSI M31-A3, 2008). The antimicrobial manufacturers’ instructions were followed when CLSI guidelines were not available. The reference strain *Staphylococcus aureus* ATCC 29213 was used for quality control for both methods.

#### Macrolide Resistance Phenotypes

All the isolates were tested for resistance only to macrolides (M phenotype) or to macrolides, lincosamides, and streptogramin-B (MLSB phenotype). The MLSB resistance, inducible (iMLSB) or constitutive (cMLSB), was checked by a double-disk test with erythromycin and pirlimycin disks. Resistance only to lincosamides (L phenotype) and to lincosamides and streptogramin-A (LSA phenotype) was described previously (Malbruny et al., 2004; Rato et al., 2013).

#### Antimicrobial Resistance Gene Detection

All the isolates with M or MLS_B_ phenotypes (Pires et al., 2005) were screened to detect *ermA*, *ermB*, and *mefA* macrolide resistance genes. Genes *tetM*, *tetT*, *tetW*, *tetL*, *tetQ tetO*, *tetK*, and *tetS* were screened among all tetracycline-resistant strains (Pires et al., 2005). All strains were tested for the presence of *linB* gene (lincosamide resistance) (Bozdogan et al., 1999). Primer sequences and amplicon expected sizes are listed in Supplementary Table 2 (Supplementary File 2). Negative results were confirmed in at least three independent assays. VSD13 and VSD19 strains were used as positive control.

### CRISPR Array Sequencing and CRISPR-Associated (Cas) Gene Screening

In this study, the genome sequence of an SDSE strain ATCC 12394 (GenBank accession number: NC_017567) was used to design specific primers to amplify the CRISPR array and *cas* genes (Supplementary Table 2 in Supplementary File 2). PCRs were performed in a thermocycler (Biometra, Göttingen, Germany) in a final volume of 25 μl containing 100 ng of bacterial DNA, 1 × PCR buffer, 2.5 mM of MgCl_2_, 0.4 mM of dNTPs, 1 U of Taq polymerase (NZYTech, Lisbon, Portugal), and 1 μM of each primer (NZYTech, Lisbon, Portugal). PCR conditions consisted of an initial denaturation cycle (95°C for 5 min), followed by 35 cycles of denaturation (95°C for 30 s), annealing (52°C for 30 s), and extension (72°C for 80 s). A final extension at 72°C for 7 min was also performed. Milli-Q water was used as a negative control in each PCR. The PCR products of CRISPR array regions were purified using the Wizard PCR Preps DNA Purification System (Promega, Madison, WI, United States). Sequencing was performed by STAB-Vida (Lisbon, Portugal) using specific primers (CRISPR Sdys For and CRISPR Sdys Rev; Supplementary Table 2 in Supplementary File 2). The obtained DNA sequences were analyzed and edited using the software CLC Bio Main Workbench. For each CRISPR array sequence, the spacers, repeats, and flanking regions were determined using CRISPRfinder and CRISPRtionary^2^. All experiments were carried out in triplicate (Grissa et al., 2007).

The similarity of the spacers, repeats, and flanking regions was analyzed using software currently available for CRISPR miner to infer a large-scale microbe–phage infection network^3^. The spacers from each strain were selected and used to BLAST (blastn with parameters settings: *e*-value < 0.01, and up to three mismatches allowed) against both bacteria and archaea virus genomes of an integrated database from the NCBI viral Genome Resource. Matches with a query cover above 90% with 100% identity were considered.

#### Prophage Identification

Prophinder (Lima-Mendez et al., 2008) was used to screen for prophage-specifying regions among the genome of SDSD, SDSE, *S. canis*, and *S. pyogenes* strains (accession numbers of genomes are available in Supplementary Table 1 in Supplementary File 1). Prophage sequences were annotated using RAST (Aziz et al., 2008). Intact prophages were manually analyzed to confirm the presence of genes indispensable to produce functional phages: left attachment site (attL); lysogeny; DNA replication; transcriptional regulation; head; tail; lysis modules; and right attachment site (attR).

### Statistical Analysis

GraphPad Prism version 7.0 was used for statistical analysis. Data analysis was performed using the chi-square method. Statistical significance was considered when *p* < 0.05.

## Results

### Bacterial Isolates and Identification

The SDSD isolates’ identification was confirmed using 16S rRNA gene sequencing. Out of 194 Gram-positive cocci, 37 isolates (19%) were identified as *S. dysgalactiae* species. Sequence analysis of the rRNA 16S from bovine SDSD isolates showed between 99.2% and 100% nucleotide identity to SDSD ATCC 2795, SDSD NCTC4670, SDSD NCTC13731, and SDSD NCTC4669 strains deposited in the NCBI Nucleotide database. UPGMA dendrogram shows the multiple alignments of rRNA 16S sequences of bovine SDSD (from this study), SDSD, SDSE, and *S. pyogenes* strains deposited in the NCBI database. See Supplementary Figure 1 (Supplementary File 3).

### Pulsed-Field Gel Electrophoresis Profiles

The 55 SDSD isolates from subclinical mastitis isolated in Portugal during 2002–2003 (collection I) and 2011–2013 (collection II) were resolved into 45 PFGE types. Epidemiologically groups or clusters (at 70% similarity) revealed two major clonal groups, including close to 90% of all bovine isolates. The macro-restriction profiles are listed in Table 1. Two or more strains share six PFGE patterns, and 31 PFGE patterns were of single isolates. Results demonstrated that there are no distinct groups among isolates of collection I and collection II.

**TABLE 1:** Characterization of bovine SDSD isolates from collection I (VSD1 to VSD11 and VSD13 to VSD19) (2002–2003) and from collection II (VSD20 to VSD55) (2011–2013).

Strain	Farm	PFGE type	Virulence genotype	Resistance genotype	Resistance phenotype
VSD1	**F**	A-1	*sagA*, *speC*, *speK*, *spd1*		tet
VSD2	**F**	J-1	*sagA*		tet
VSD3	**J**	L-1	*sagA*	*ermA/TR*, *tetM*	cMLSB + tet
VSD4	**E**	M-1	*sagA*, *speC*, *speK*, *spd1*	*linB*, *tetM*	L + tet
VSD5	**E**	F-1*	*sagA*, *sdn*	*tetM*	tet
VSD6	**I**	D-1	*sagA*, *speK*, *speL*	*tetM*	tet
VSD7	**D**	G-1	*sagA*, *speM*, *sdn*	*tetO*	tet
VSD8	**G**	C-1	*sagA*, *sdn*		tet
VSD9	**B**	N-1	*sagA*	*ermB*, *tetO*	cMLSB + tet
VSD10	**G**	C-1	*sagA*, *sdn*		tet
VSD11	**F**	B-1	*sagA*, *speC*, *speK*, *spd1*	*linB*, *tetO*	L + tet
VSD13	**G**	I-1	*sagA*, *speC*, *speK*, *speL*, *speM*, *spd1*	*ermB*, *tetO*	cMLSB + tet
VSD14	**F**	C-2	*sagA*,	*ermB*, *tetO*	cMLSB + tet
VSD15	**J**	D-1	*sagA*, *speK*, *speL*	*tetM*	tet
VSD16	**B**	A-2	*sagA*, *speC*, *speK*, *spd1*		tet
VSD17	**C**	E-1	*sagA*		tet
VSD18	**I**	D-1	*sagA*, *speK*, *speL*	*tetM*	tet
VSD19	**E**	M-1	*sagA*, *speC*, *speK*, *spd1*	*linB*, *tetM*	L + tet
VSD20	**S**	O-1	*sagA*, *speL*	*ermB*, *tetO*	cMLSB + tet
VSD21	**Q**	P-1	*sagA*, *speL*, *speM*	*ermB*, *tetM*, *tetO*	cMLSB + tet
VSD22	**Q**	Q-1	*sagA*, *speM*	*tetM*, *tetO*	tet
VSD23	**V**	R-1	*sagA*, *speM*, *sdn*	*tetM*, *teto*, *tetK*	tet
VSD24	**S**	S-1	*sagA*, *speC*, *speK*, *spd1*		
VSD25	**N**	T-1	*sagA*, *sdn*	*tetM*, *tetK*	tet
VSD26	**N**	V-1	*sagA*, *speK*	*tetM*, *tetO*	tet
VSD27	**M**	X-1	*sagA*	*tetM*, *tetO*	tet
VSD28	**X**	Z-1	*sagA*, *sdn*	*tetO*, *tetK*	tet
VSD29	**M**	K-1	*sagA*, *speM*	*tetM*, *tetO*	tet
VSD30	**T**	Y-1	*sagA*, *speL*, *speM*	*tetM*	tet
VSD31	**M**	W-1	*sagA*	*tetM*, *tetO*	Tet
VSD32	**O**	AA-1	*sagA*, *speK*, *sdn*	*tetO*	Tet
VSD33	**O**	AA-1	*sagA*, *speK*, *sdn*	*tetO*	Tet
VSD34	**P**	AB-1	*sagA*, *speM*	*tetM*, *tetO*, *tetK*	Tet
VSD35	**N**	Z-1	*sagA*, *speL*, *speM*, *sdn*	*tetO*, *tetK*	Tet
VSD36	**M**	W-1	*sagA*, *speL*, *speM*	*tetM*, *tetO*	Tet
VSD37	**O**	AC-1	*sagA*, *speK*, *sdn*	*tetO*	
VSD38	**U**	W-1	*sagA*	*tetM*, *tetO*	tet
VSD39	**U**	AD-1	*sagA*	*tetM*, *tetO*	tet
VSD40	**R**	AE-1	*sagA*, *sdn*	*tetO*, *tetK*	tet
VSD41	**M**	W-1	*sagA*	*tetM*, *tetO*	tet
VSD42	**M**	W-1	*sagA*	*tetM*, *tetO*	tet
VSD43	**M**	AF-1	*sagA*, *speK*, *sdn*	*tetM*, *tetO*	tet
VSD44	**S**	AG-1	*sagA*, *speC*, *speK*, *spd1*	*tetO*	tet
VSD45	**U**	AH-1	*sagA*	*tetM*	tet
VSD46	**U**	AI-1	*sagA*	*tetM*	tet
VSD47	**M**	AJ-1	*sagA*, *speC*, *speK*, *spd1*	*tetM*, *tetO*	tet
VSD48	**M**	AJ-1	*sagA*, *speC*, *speK*, *spd1*	*tetM*, *tetO*	tet
VSD49	**M**	AL-1	*sagA*, *speC*, *spd1 speK*	*tetM*, *tetO*	tet
VSD50	**R**	V-1	*sagA*, *speL*, *speK*	*tetM*, *tetO*	tet
VSD51	**M**	AM-1	*sagA*, *speL*, *speM*	*tetM*, *tetO*	tet
VSD52	**O**	AN-1	*sagA*		
VSD53	**U**	AI-1	*sagA*	*tetM*, *tetO*	tet
VSD54	**R**	V-1	*sagA*, *speL*, *speK*	*tetM*, *tetO*	tet
VSD55	**R**	AO-1	*sagA*, *speM*, *sdn*	*tetM*, *tetO*	tet
VSD56	**X**	AP-1	*sagA*, *speM*, *sdn*		

SDSD, *Streptococcus dysgalactiae* subsp. *dysgalactiae*; PFGE, pulsed-field gel electrophoresis. *cfr9I patterns.

### Phylogenetic Analysis

**FIGURE 1:**
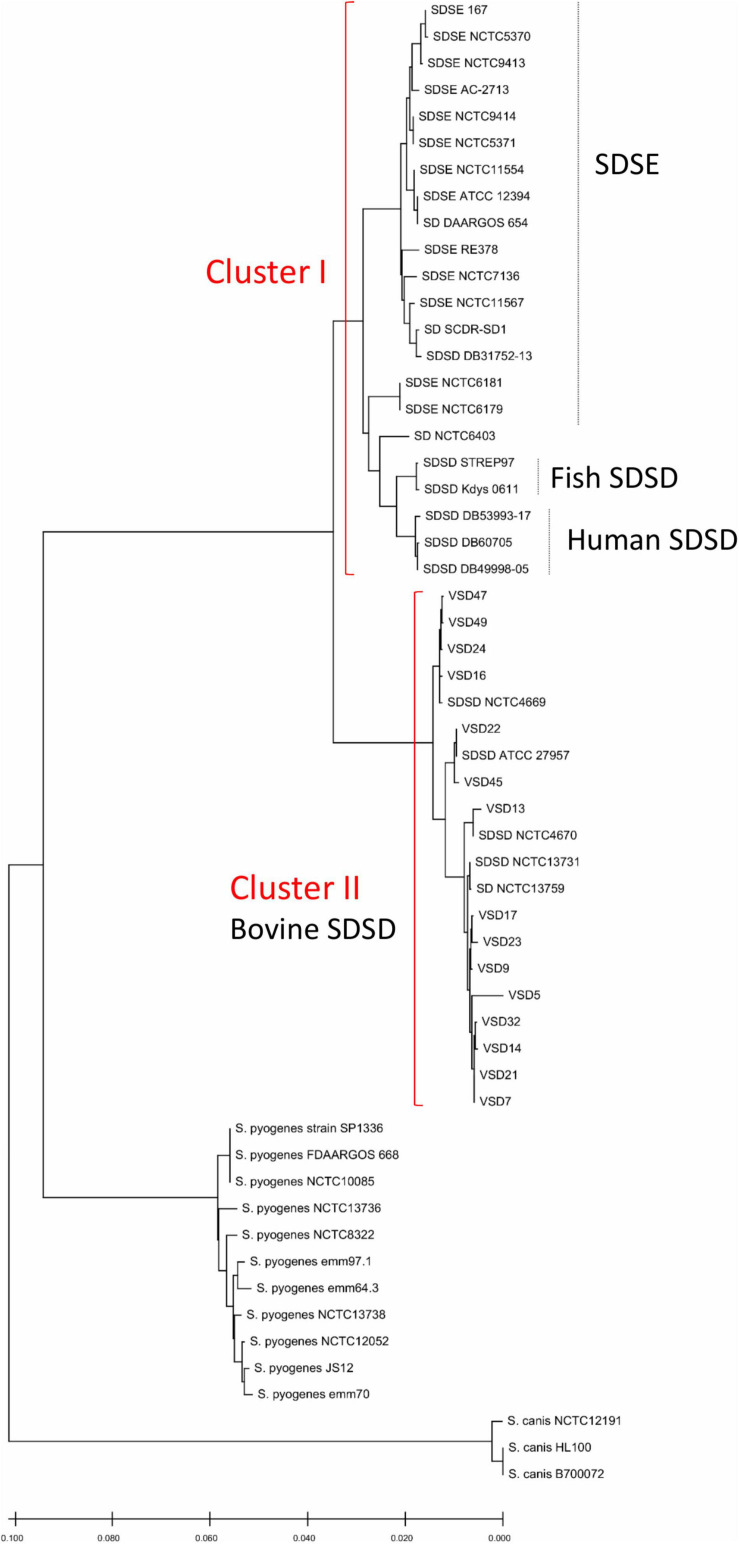
Phylogenetic analysis based on concatenated sequences of seven housekeeping genes (*gki*, *gtr*, *murI*, *mutS*, *recP*, *xpt*, and *atoB*) showing the position of the 15 bovine SDSD isolates and sequences of strains available in the National Center for Biotechnology Information (NCBI) database, including SDSD strains (*n* = 12), SDSE strains (*n* = 15), *Streptococcus pyogenes* strains (*n* = 11), *Streptococcus canis* strains (*n* = 3). VSD, Vet *S. dysgalactiae* subsp. *dysgalactiae*. Bovine SDSD isolates: ATCC 27957; NCTC13731; NCTC4669; NCTC4670 and VSDs. Human SDSD strains: DB31752-13; DB49998-05; DB60705-15, and DB53993-17. Fish SDSD strain: SDSD Kdys0611. SDSE, *S. dysgalactiae* subsp. *equisimilis*. The phylogenetic relationships were inferred using the minimum evolution method, and phylogenetic distances were estimated using the maximum composite likelihood method using the MEGA (Kumar et al., 2018). The tree is drawn to scale, with branch lengths in the same units as those of the evolutionary distances used to infer the phylogenetic tree.

Alignment of concatenated sequences of seven housekeeping genes (*gki*, *gtr*, *murI*, *mutS*, *recP*, *xpt*, and *atoB*) was created using the standard parameters of ClustalW. Then a minimum evolution tree was obtained from sequences from bovine SDSD isolates (determined in this study) and sequences of a selection of SDSD, SDSE, *S. pyogenes*, and *S. canis* strains available in the NCBI database (Supplementary Table 1 in Supplementary File 1 and Figure 1).

*Streptococcus canis* and *S. pyogenes* constitute monophyletic clusters. The *S. dysgalactiae* cluster was divided into two groups: cluster 1—human and fish SDSD strains, and SDSE; cluster 2—bovine SDSD isolates. Data show that human SDSD strains are closer to SDSE than bovine SDSD isolates. Interestingly, this phylogenetic relationship between SDSD and SDSE obtained by MLSA does not reflect the relationship based on the 16S rRNA sequences (Figure 1 and Supplementary Figure 1 in Supplementary File 3). The accession numbers of *gki*, *gtr*, *murI*, *mutS*, *recP*, *xpt*, and *atoB* genes are present in Supplementary Table 3 (Supplementary File 5).

### Virulence Genes

All the 37 bovine SDSD isolates (collection II), screened by PCR, carry at least one of the following superantigens (SAgs) genes (*speC*, *speK*, *speL*, and *speM*), DNase (*spd1*), streptodornase (*sdn*), or SLS (*sagA*). None of the strains carry *speA*, *speB*, *speF*, *speG*, *spegg*, and *speH* SAgs genes; *smeZ* mitogenic exotoxin gene; and *sagB*, *sagE*, and *sagI* genes.

The results showed that the SAgs genes were unevenly distributed among all the 37 bovine SDSD isolates (see Table 1). The screening revealed the following distribution: *speC*, 20%; *speK*, 40%; *speL*, 20%; *speM*, 23.6%; *spd1*, 20%; and *sdn*, 27.2% positive isolates. Transcriptional analysis showed that all virulence genes were transcribed in all SDSD isolates that carried them.

Similar virulence gene patterns were found among bovine SDSD from collection I (Rato et al., 2010, 2011) and II. No relationships were found between virulence profiles and the farm or macro-restriction patterns (see Table 1).

**FIGURE 2:**
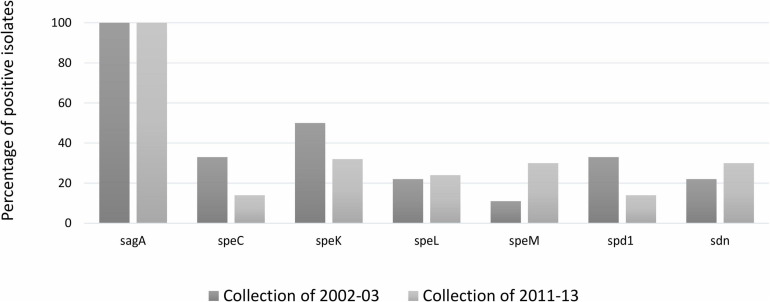
Distribution (%) of group A *Streptococcus pyogenes* virulence gene among *Streptococcus dysgalactiae* subsp. *dysgalactiae* collections isolate in 2002–2003 and 2011–2013. *speC*, *speK*, *speL*, *speM*: encode streptococcal pyrogenic exotoxins; *spd1*: DNase; *sdn*: streptodornase (DNase). The statistical significance of the data was determined by a chi-square test (χ^2^), where a probability value (*p*) ≤ 0.05 was considered as statistically significant.

The comparison of the distribution of virulence genes between SDSD isolates from collection I and II is shown in Figure 2. Chi-square statistical analysis revealed that each independent virulence gene proportion was not statistically different between collections.

The comparison of the homologous sequences of bovine SDSD SAgs genes with *S. pyogenes* pyrogenic exotoxin gene from the NCBI databases, showed high identity: >98% identity for *speC*; >97% for *speK*; >98% for *speM*; and >97% for *speL*. In addition, a high identity was also found for DNAses *spd1* (>98%) (Supplementary Tables 4–7 in Supplementary File 4).

For a comparative analysis, the presence of virulence genes in the genomes of the streptococci listed in Supplementary Table 1 (Supplementary File 1) was analyzed. None of the *speB*, *speC*, *speF*, *speG*, *speH*, *speK*, *speL*, *speM*, and *smeZ* virulence genes were detected among human SDSD or SDSE strains. Streptodornase (*sdn*) was found among the human SDSE strains (3/15), *S. pyogenes* strains (2/15), bovine SDSD isolates (5/20), but it was not detected in human SDSD or *S. canis* strains. *spegg* gene was found in SDSE, *S. pyogenes*, and human SDSD, but not in bovine SDSD; and the *smeZ* was found only among *S. pyogenes* strains (Supplementary Figure 2 in Supplementary File 3). The nucleotide sequences of *spegg* gene in human and fish SDSD strains revealed >99% identity with its homolog in *S. pyogenes* from the NCBI database.

**FIGURE 3:**
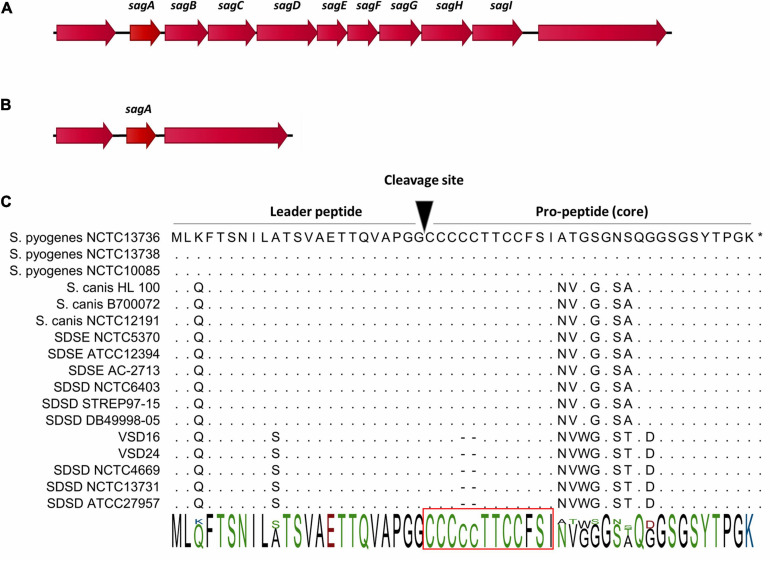
Gene cluster organization of streptolysin S (SLS) operon and precursor peptide sequences of SLS cytolysins (SagA). **(A)** Organization of the SLS operon in *Streptococcus dysgalactiae* subsp. *equisimilis* (SDSE), human and fish *S. dysgalactiae* subsp. *dysgalactiae* (SDSD), *Streptococcus pyogenes*, and *Streptococcus canis*. The operon encoding SLS includes the prepropeptide structural gene (*sagA*), followed by eight genes responsible for the conversion of SagA into SLS (*sagBCD*), transport across the membrane (*sagFGHI*), and accountable for leader cleavage (*sagE*) (Datta et al., 2005). **(B)** Organization of the genomic region flanking *sagA* in bovine SDSD isolates. **(C)** Alignment of SagA peptide, the precursor of SLS. SLS core region possesses a highly conserved N-terminus, while the C-terminus is more variable. The putative leader peptide cleavage site is shown. Red rectangle, minimal core region required for hemolytic activity of SLS in *S. pyogenes*. STAB-Vida performed sequencing of sagA. Deduced amino acid sequences from this bovine allele were compared with sequences from the National Center for Biotechnology Information (NCBI) database and were analyzed with the CLC-bio Main Workbench sequence alignment tool (QIAGEN, Netherlands).

In addition, all the bovine SDSD isolates from collection I and the genomes listed in Supplementary Table 1 (Supplementary File 1) were tracked for the presence of the SLS operon. The comparative analysis showed that of the nine genes that are part of the SLS operon, the bovine SDSD isolates only had *sagA* gene (Table 1), while the SDSE, human and fish SDSD, *S. pyogenes*, and *S. canis* strains harbored the complete operon (Figures 3A,B and Supplementary Figure 2 in Supplementary File 3). Figure 3C shows the amino acid sequence of SagA protein of the bovine SDSD isolates deduced through *in silico* analysis, compared with SDSE, SDSD, *S. canis*, and *S. pyogenes* sequences. The alignment of SagA peptide demonstrated that the leader-peptide region is highly conserved, while the pro-peptide region is more variable. In this last region, the bovine SDSD isolates present a deletion of two cysteine residues.

### Antimicrobial Resistance Patterns

**FIGURE 4:**
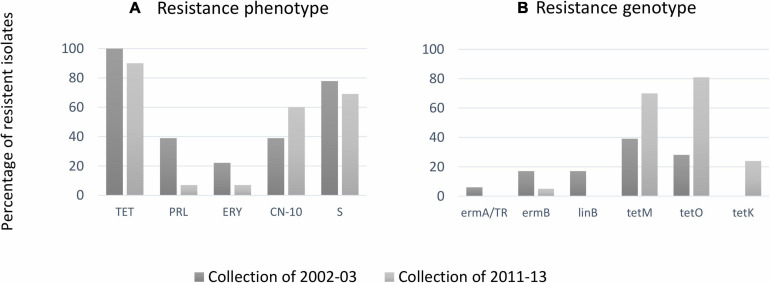
**(A)** Percentages of antimicrobial resistance among bovine *Streptococcus dysgalactiae* subsp. *dysgalactiae* (SDSD) isolates from the present study against erythromycin (ERY), gentamicin (CN), pirlimycin (PRL), streptomycin (S), and tetracycline (TET). Resistance was not observed against amoxicillin–clavulanic acid (AMC), cefazolin (KZ), chloramphenicol (CHL), penicillin (P), rifaximin (RAX), and vancomycin (VA). **(B)** Distribution resistance genotypes among bovine SDSD: *ermA* and *ermB*: macrolide resistance genes; *linB*: lincosamide resistance gene; *tetK*, *tetM*, and *tetO*: resistance to tetracycline genes. The statistical significance of the data was determined by a chi-square test (χ^2^), where a probability value (*p*) ≤ 0.05 was considered as statistically significant.

Among the 37 bovine isolates from collection II, the highest antimicrobial resistance patterns were observed for tetracycline (90%), followed by streptomycin (69%) and gentamicin (60%). None of the streptococcal isolates tested showed resistance to amoxicillin/clavulanic acid, cefazolin, cefoperazone, chloramphenicol, penicillin, rifaximin, and vancomycin (Table 1 and Figure 4). Two isolates resistant to pirlimycin and erythromycin presented the cMLSB phenotype (Table 1), while the phenotype M (erythromycin-resistant only) was not detected. The two strains showed the cMLS_B_ phenotype (constitutive MLSB). Strains of the cMLSB phenotype harbored *ermB* gene, while *ermA* and *mefA* (macrolide resistance genes) were not detected (Table 1). The resistance to tetracycline was associated with *tetM*, *tetK*, and *tetO* (*tetS*, *tetT*, *tetW*, *tetL*, and *tetQ* negative). Figure 4 compares the resistance phenotypes distribution and genotypes among the 55 bovine SDSD isolates from collection I (Rato et al., 2013) and collection II. Chi-square statistical analysis revealed that each independent resistance phenotype proportion was not statistically different between collections. However, statistically significant differences were observed between *tetM*, *tetK*, and *tetO*.

### CRISPR System Analysis and Prophage Identification

**FIGURE 5:**
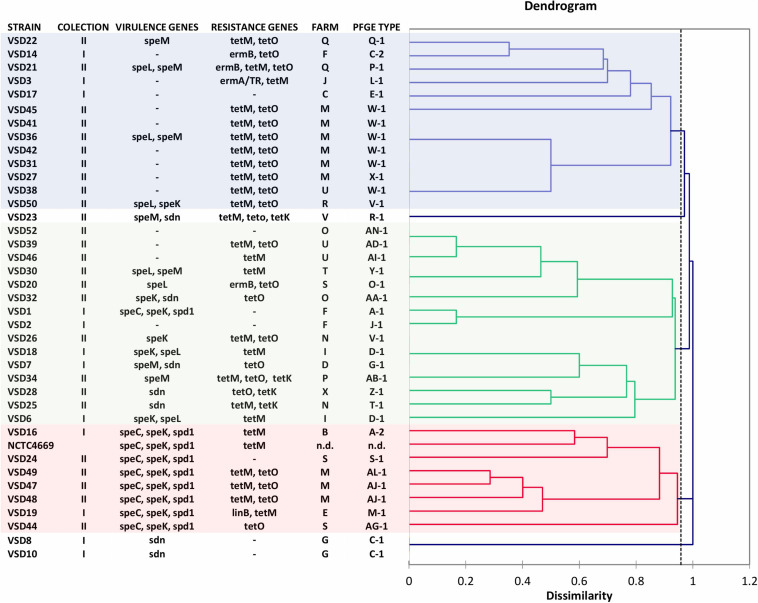
Dendrogram based on spacer CRISPR IIA cluster of *Streptococcus dysgalactiae* subsp. *dysgalactiae* isolates of mastitis in cattle collected in 2002–2003 (Rato et al., 2010, 2011, 2013) (VSD1 to VSD19—collection I) and 2011–2013 (VSD20 to VSD56—collection II). *Streptococcus pyogenes* phages encode genes *speC*, *speK*, *speL*, *speM*, *spd1*, and *sdn*. The dendrogram was obtained by means of Dice coefficient and the agglomerative clustering of the unweighted pair group method with arithmetic mean (UPGMA).

SDSD isolates from collection I of 2002–2003 (*N* = 18) and collection II of 2011–2013 (*N* = 37) were analyzed by sequencing the CRISPR loci. Overall, the analysis of CRISPR array discloses a high intrinsic diversity among bovine SDSD understudy, demonstrating the distinguish groups among isolates from both collections (Figure 5).

Among the 55 bovine SDSD isolates analyzed for the CRISPR array, 49 were PCR positive (but only 41 strains were CRISPR spacer positive; see Supplementary File 6), and four were PCR negative. For two strains, the amplified product showed high homology (>99%) with ISSdy transposase (ORF A, GenBank accession: VDZ39966.1 and ORF B, GenBank accession: VDZ39965.1).

A high degree of direct repeat (DR) sequence (5′GTTTTAGAGCTGTGCTGTTTCGAATGGTTCCAAAAC′3) conservation was observed among bovine SDSD isolates. The DR showed sequence similarity to typical repeat sequences of other streptococci species, such as *S. agalactiae* with 100% of identity (Lier et al., 2015), *Streptococcus thermophilus* with an average identity of about 85% (Hu et al., 2020), and *S. pyogenes* with >95% of identity (Nozawa et al., 2011).

A total of 158 spacers were identified among the 41 CRISPR spacer positive strains, of which 80 (50%) were found to be unique. The number of spacers in each CRISPR array was eight on average, ranging from a maximum of 26 to a minimum of 2 (Supplementary Tables 8, 9 in Supplementary File 6). Most strains (39 of 41) shared at least one spacer. Only VSD4 and VSD13 did not share spacers; these strains harbored six and three unique spacers, respectively. The SDSD VSD5, VSD9, VSD11, VSD29, VSD33, VSD37, and VSD40 isolates were CRISPR spacer negative (Supplementary File 6).

Three major groups were observed based on the common spacer sequences or absence of spacers (Figure 5 and Supplementary Table 9 in Supplementary File 6). Some old spacers were detected in bovine SDSD that harbored the same combinations of SAgs. For example, the 41, 42, 62, 63, 64, 65, 66, 118, 119, 120, and 121 spacers were detected among isolates from collection I (VSD16 and VSD19) and collection II (VSD24, VSD47, VSD48, and VSD49) harboring *speC*, *speK*, and *spd1* genes (Figure 5 and Supplementary File 6). Some of these spacers were also detected in SDSD NCTC4669 (isolated in 1935).

About 72% (114/158) spacers matched with sequences present in the genome of *S. pyogenes*, *S. agalactiae*, *S. canis*, and *S. dysgalactiae* with a similarity >95. A BLAST was carried out to determine if the spacers had an origin in bacteriophage. Sequences above 95% of identity, in both query coverage and percent identity, were considered. Of the 158 spacer sequence, 29 (18.4%) showed high identity (bit scores ≥ 50) with prophage regions. These regions correspond to phage isolated from *S. pyogenes* and phages belonging to others species, namely, *S. agalactiae* str. ILRI005; *Listeria* phage B054; *Flavobacterium* phage 11b; *Streptococcus* phage SpSL1; *Synechococcus* phage S-CAM7; *Bacillus* virus IEBH; and *Bacillus* virus 250 (Table 2). About 28% of the sequence spacers of SDSD were not identified in the NCBI database.

**TABLE 2:** Identification and nucleotide sequence of the bacteriophage spacers in a CRISPR array of SDSD.

Spacer	Sequence	Bacteriophage	SDSD strain
**sp4**	ATAAATTTTTGTTGTAGCGAGTCTTACCGT	*Streptococcus* phage phi3396	NCTC4669
**sp6**	ATTGAGAATAGAGCGATATAAACAGGAGAA	Temperate phage phiNIH1.1	NCTC4669, VSD16
**sp7**	TTAGGCGCCAGCGTTAAAGAGGTGTTTGCT	*Streptococcus* phage 315.6	NCTC4669
**sp11**	TTGTTTTTGGACTTGCGGTTAATCATAAAA	*Streptococcus* phage 315.2	NCTC4669, VSD16
**sp14**	AGTGTTACTTGAACCAACACCCCATCTAAG	*Streptococcus* phage 315.6 or S. phage P9	NCTC4669, VSD16
**sp15**	AGTGTTACTTGAACCAACACCCCATCTAAG	*Streptococcus* phage P9	NCTC4669
**sp17**	TTAGGCGCCAGCGTTAAAGAGGTGTTTGCT	*Streptococcus* phage P9	NCTC4669, VSD24
**sp25**	TAGCGACAATTTAATAATAGCTTCGATTTT	*Streptococcus* phage 315.5	VSD1, VSD2, VSD32, VSD46, VSD55
**sp30**	TCATGCACCTCAATGTCCTTACGCATCTCC	*Streptococcus* phage phi3396	VSD1, VSD2
**sp35**	AGCATTTCTGCAAACTCTGCCATGTATTGA	*Streptococcus* phage 315.1	VSD10, VSD8
**sp36**	CAAAGCCCCTCAAAATGCAACACAGTAATG	*Streptococcus* phage 315.3	VSD10, VSD8
**sp49**	AGCCTAGCAAGCATAAGTAATCGCTAATGG	*Streptococcus* phage 315.1	VSD14, VSD22, VSD45
**sp50**	CCTTTTGCACGGCTTCGCCCATAGCGATTT	*Streptococcus* phage 315.2	VSD14, VSD21, VSD22, VSD45
**sp52**	TATATCATCTCCTAGTGATAAACCTGCCAG	*Streptococcus* phage 315.3	VSD14, VSD21, VSD22, VSD3
**sp53**	AGAACGTATTGACGGTATCGTTAAAGTAAC	*Bacillus* virus IEBH or *Bacillus* virus 250	VSD14, VSD22, VSD3
**sp55**	AACCATTGCGTAGTCATAATCATCAAGCAT	*Streptococcus* phage 315.5	VSD14, VSD3
**sp58**	TTAAAGGCAGATACGTTCTGATATGGGGCT	*Listeria* phage B054	VSD14, VSD21, VSD22, VSD27, VSD31, VSD36, VSD38, VSD41
**sp81**	GTTGATTGATCAACAGTTGGTTGAGACAAA	*Streptococcus* phage 315.5 or S. phage phi3396	VSD19, VSD47, VSD48, VSD49
**sp88**	ACTAACCACAAGCAAGGTTGCGACCCTTGT	Temperate phage phiNIH1.1 or S. phage 315.2	VSD20, VSD30, VSD32, VSD39, VSD46, VSD53, VSD55
**sp103**	TTTCCCTATGATAAACCTTTAATAAAGTGT	Temperate phage phiNIH1.1 or S. phage 315.4	VSD25
**sp104**	AAAACTACAAACGCTTGCTGATAAAACCAA	*Streptococcus* phage phi3396	VSD25
**sp105**	TAATAAGCATTTTGATTTAGCTATTGTTG	*Flavobacterium* phage 11b or S. phage SpSL1	VSD25, VSD35
**sp106**	CCATTAGCAATAGCAGGATTA	*Synechococcus* phage S-CAM7	VSD25
**sp116**	AGAATAGAGAAAAAAGAGATATTTTTTGAT	Temperate phage phiNIH1.1 or S.s phage 315.1	VSD32
**sp138**	TCAACCACATCTTTATAAGACATCTCAAGC	*Streptococcus* phage 315.3	VSD45
**sp139**	AGCCTCGCAAGCATAAGTAATCGCTAATGG	*Streptococcus* phage 315.5	VSD45
**sp140**	GCTTGGGACGTTGGATTGTTGATTTTTATT	*Streptococcus* phage 315.4	VSD45
**sp149**	AGATTTGCCTTCATCAGCGATGTTTTTGTT	*Streptococcus* phage 315.5	VSD47, VSD48, VSD49, VSD16
**sp158**	AAAATTTATTGATGGCGAATCGGTGCCAGA	*Streptococcus* phage 315.3	VSD49, VSD16

*SDSD, *Streptococcus dysgalactiae* subsp. *dysgalactiae*.*

Due to the similarity of some spacers between contemporary bovine SDSD isolates and SDSD NCTC4669 (isolated in 1935, the oldest documented with sequenced genome) (Chanter et al., 1997), we analyze the sequences adjacent to a CRISPR array of this oldest strain. The genetic map of this region consisted of only four *cas* genes (*cas9*, *cas1*, *cas2*, and *csn2*), corresponding to the CRISPR type IIA system. Additionally, the ISSdys transposase gene was found in type IIA system.

The genes associated with the type IIA CRISPR system were searched among the 55 bovine SDSD isolates (collection I and II). All bovine SDSD isolates were PCR positive to *cas9*, *cas1*, *cas2*, and *csn2* genes. Additionally, two of the 55 strains were PCR positive for ISSdys transposase.

To elucidate the relationship between the prophages and CRISPR systems among SDSD strains, we analyzed CRISPR array, CRISPR-associated (Cas) proteins, and prophage structure using complete genome sequences of SDSD listed in Supplementary Table 1 (Supplementary File 1). For a comparative analysis, the complete genome of SDSE, *S. canis*, and *S. pyogenes* was also analyzed.

The CRISPR/Cas loci of the SDSD and SDSE strains analyzed were classified as type I-C and/or type II-A (Table 3). SDSD and SDSE genomes showed a high number of spacers when compared with *S. pyogenes*. While CRISPR arrays of *S. pyogenes* have between 0 and 10 spacers, SDSD and SDSE can have more than 40 spacers (Table 3). No CRISPR system was observed in 15% and 42% of the SDSE and *S. pyogenes* genomes, respectively, while in all SDSD strains, CRISPR/Cas system is present.

**TABLE 3:** Identification of CRISPR/Cas systems and prophage or prophage-like regions.

Strain	CRISPR system | no.	Prophage
	spacer	regions*
**SD FDAARGOS 654**	Type IA | 12; Type IIC | 19	1| 5
**SD NCTC13759**	Type IA | 3	2| 8
**SD NCTC6403**	Type IIC | 9	1| 2
**SD SCDR-SD1**	Type IA | 15; Type IIC | 20	0| 3
**SDSD ATCC 27957**	Type IA | 3	2| 6
**SDSD DB31752-13**	Type IA | 33; Type IIC | 11	0| 5
**SDSD DB49998-05**	Type IIC | 28	0| 4
**SDSD DB53993-17**	Type IA | 3	0| 2
**SDSD DB60705-15**	Type IIC | 42	3| 8
**SDSD Kdys0611**	Type IIC | 22	1| 12
**SDSD NCTC13731**	Type IA | 3	2| 8
**SDSD NCTC4669**	Type IA | 14; Type IIC | 22	3| 13
**SDSD NCTC4670**	Type IICA | 4	2| 11
**SDSD STREP97-15**	Type IIC | 7	3| 20
**SDSE NCTC11554**	Type IA | 13; Type IIC | 34	1| 5
**SDSE NCTC5370**	No detected	1| 4
**SDSE NCTC5371**	Type IIC | 42	1| 5
**SDSE NCTC6179**	Type IA | 3; Type IIC | 9	2| 4
**SDSE NCTC6181**	Type IA | 3; Type IIC | 9	2| 6
**SDSE NCTC7136**	Type IA | 25; Type IIC | 15	3| 4
**SDSE NCTC9413**	Type IIC | 11	1| 5
**SDSE NCTC9414**	Type IIC | 42	1| 6
**SDSE 167**	No detected	1| 4
**SDSE AC-2713**	Type IA | 15; Type IIC | 20	1| 8
**SDSE ATCC 12394**	Type IA | 30; Type IIC | 26	0| 4
**SDSE GGS 124**	Type IIC | 19	1| 4
**SDSE RE378**	Type IA | 14; Type IIC | 8	0| 5
***Streptococcus canis* B700072**	Type IIC | 18	0| 4
***S. canis* NCTC12191**	Type IA | 8; Type IIC | 2	2| 3
***S. canis* HL_100**	Type IA | 12; Type IIC | 21	1| 4
***Streptococcus pyogenes* emm64.3**	No detected	1| 3
***S. pyogenes* emm70**	No detected	1| 3
***S. pyogenes* emm97.1**	No detected	1| 1
***S. pyogenes* FDAARGOS_668**	Type IA | 7; Type IIC | 3	2| 4
***S. pyogenes* JS12**	Type IIC | 3	1| 2
***S. pyogenes* NCTC10085**	Type IA | 4; Type IIC | 3	2| 2
***S. pyogenes* NCTC12052**	Type IIC | 3	3| 7
***S. pyogenes* NCTC12696**	Type IIC | 0	3| 6
***S. pyogenes* NCTC13736**	No detected	4| 5
***S. pyogenes* NCTC13738**	No detected	3| 5
***S. pyogenes* NCTC8322**	Type IA | 7; Type IIC | 10	3| 4
***S. pyogenes* SP1336**	Type IA | 6; Type IIC | 0	2| 4

SDSE, *Streptococcus dysgalactiae* subsp. *equisimilis*; SDSD, *S. dysgalactiae* subsp. *dysgalactiae*.

*Complete prophages | total of putative prophage regions. Bovine SDSD isolates: ATCC 27957, NCTC13731, NCTC4669, and NCTC4670. Human SDSD strains: DB31752-13, DB49998-05, DB60705-15, and DB53993-17. Fish SDSD strain: SDSD Kdys0611 and SDSD STREP97-15.

The total number of prophage regions obtained through *in silico* analysis was 228 in 42 genomes, with an average of 5.45 prophages per genome (Table 3). *S. pyogenes* had the highest number of complete prophages per genome, i.e., 2.1, followed by SDSD with two complete prophages per genome. SDSE and *S. canis* had 1.15 and 1 complete prophages per genome.

The SDSD STREP97-15 strain presented the highest number of prophage regions (20); however, only three prophage regions appear to be complete. Among human SDSD strains, only SDSD DB60705-15 harbored complete prophages (3). SDSD DB31752-13, DB49998-05, and DB53993-17 did not harbor any complete prophages. On the other hand, all bovine and fish SDSD strains harbored complete prophages. The complete prophage genomes of the SDSD strains are listed in Table 4. Overall, there was no statistical difference between the total number of prophage regions in type IC SDSD strains and type IIA SDSD strains.

**TABLE 4:** Prophage regions identified by computational tools for the genome of *Streptococcus dysgalactiae* subsp. *dysgalactiae* strains.

Strain	Region length	Score	Total proteins	Phage most common	GC%
NCTC4669	54.7 kb	140	82	Strept T12 (NC_028700)	37.71
	49.9 kb	150	65	Strept 315.4 (NC_004587)	38.96
STREP97-15	50 kb	130	56	Strept 315.5 (NC_004588)	37.96
	43.4 kb	150	66	Strept phi3396 (NC_009018)	36.85
	42.1 kb	150	51	Strept phiNJ2 (NC_019418)	38.29
NCTC4670	55.5 kb	150	84	Strept T12 (NC_028700)	38.44
	49.1 kb	130	82	Strept T12 (NC_028700)	37.88
ATCC 27957	62.5 kb	130	79	Strept 315.3 (NC_004586)	38.80
	58.8 kb	150	76	Strept 315.3 (NC_004586)	38.68
NCTC13731	79.2 kb	150	104	Strept T12 (NC_028700)	38.32
	50 kb	140	68	Strept 315.4 (NC_004587)	39.59
NCTC13759	79.2 kb	150	104	Strept T12 (NC_028700)	38.32
	50 kb	140	67	Strept 315.4 (NC_004587)	39.59
DB60705-15	34.1 kb	140	49	Strept A25 (NC_028697)	41.13
	36.6 kb	150	55	Strept phiNJ2 (NC_019418)	37.20
	27.4 kb	140	31	Escher RCS47 (NC_042128)	34.78
Kdys0611	36 kb	100	24	Geobac GBSV1 (NC_008376)	40.00

*Bovine SDSD isolates: ATCC 27957, NCTC13731, NCTC4669, and NCTC4670. Human SDSD strains: DB49998-05 and DB60705-15. Fish SDSD strain: SDSD Kdys0611 and SDSD STREP97-15.*

*GC, guanine–cytosine; SDSD, *S. dysgalactiae* subsp. *dysgalactiae*.*

## Discussion

In the present study, the MLSA was performed according to the method previously described (McMillan et al., 2010). In a previous study, the phylogeny of *S. canis*, *S. dysgalactiae*, *Streptococcus equi*, and *S. pyogenes* species based on MLSA (*map*, *pfl*, *ppaC*, *pyk*, *rpoB*, *sodA*, and *tuf* genes) was reconstructed according to this analysis; *S. dysgalactiae* includes two separate clusters corresponding to the “*dysgalactiae*” and “*equisimilis*” subspecies (Jensen and Kilian, 2012). Surprisingly, our results show the separation of bovine SDSD from human SDSD strains in different clusters, the latter being closer to the SDSE (Figure 1).

In this study, we searched for the presence of different *S. pyogenes* virulence genes (encoded by MGE or either chromosomal) among bovine SDSD isolates, namely, genes encoding DNAses, SAgs, and SLS.

Some of the SAgs genes are phage- and transposon-associated among *S. pyogenes* strains (Proft and Fraser, 2016). Previous studies have related some of these genes to an increase in the pathogenic potential of SDSD isolates (Rato et al., 2010, 2011). Our results show that the SAgs genes (*speC*, *speK*, *speL*, and *speM*) and *spd1* are unevenly distributed among bovine SDSD isolates (from collection I and collection II) but not among human and fish SDSD strains (Table 1 and Supplementary Figure 2 in Supplementary File 3).

The *speC* and *spd1* (initially identified in the M1 phage) genes were always detected together, and it was also found to be associated with *speK* (initially identified in the M3 phage) among bovine SDSD isolates, indicating the probable poly-lysogeny, as described for *S. pyogenes* (Giovanetti et al., 2015). We also found the *speC*–*spd1*–*speK* genes in the Strept T12 NC_028700 prophage of the bovine SDSD NCTC4669 strain.

Alignment of *speC*, *speK*, *speL*, *speM*, and *spd1* sequence of bovine SDSD against the GenBank database, using BLAST, revealed a nucleotide identity of 97% to >99% with the homologous *S. pyogenes* virulence (Supplementary File 5). Although the genomes of the bovine SDSD isolates from collection I and II had not been sequenced, the high homology of SAgs and the presence of these genes in prophage regions among *S. pyogenes* could indicate that similar phages (or even the same) are shared among the bovine SDSD and *S. pyogenes*. The high identity of the SAgs and the different habitats of SDSD (animals) and *S. pyogenes* (human pathogen) may suggest that the HGT events occurred before habitat separation.

We analyzed the presence of virulence genes among streptococcal genomes listed in Supplementary Table 1 (Supplementary File 1; Supplementary Figure 2 in Supplementary File 3). We found the streptococcal pyrogenic exotoxin G gene (*spegg*) in SDSE, *S. pyogenes*, and human SDSD, but not in bovine SDSD. The presence of *spegg* has been documented in fish SDSD strains (Abdelsalam et al., 2010); however, to our knowledge, the presence of this gene has not been reported so far among human SDSD strains. Nucleotide sequences from the human and fish SDSD strains revealed >99% identity with the homologous *spegg* of *S. pyogenes* from the NCBI database. To date, there is no evidence of HGT of the *spegg*; so its absence may mean the loss of this in bovine SDSD isolates common ancestor.

SDSD (α-hemolytic) and SDSE (β-hemolytic) have been distinguished based on their hemolytic properties on blood agar in routine laboratory practice. The hemolytic activity is mainly attributed to SLS production among the pyogenic group of streptococci.

In this study, the results showed the loss of *sag*B-I genes among bovine SDSD isolates. At the same time, the presence of the SLS operon was observed in human and fish SDSD, SDSE, *S. pyogenes*, and *S. canis* (β-hemolytic strains). Our data are the first to suggest that hemolytic differences between human SDSD strains (β-hemolytic) and bovine SDSD isolates (α-hemolytic) may be related to the loss of SagB-I and mutation of *sagA* gene observed in bovine SDSD isolates (Figure 3). Likely, the exact incidence of SDSD human infections on a global basis can be underestimated, mainly because of the failure to distinguish SDSD from SDSE strains in clinical laboratories.

Even though SDSD remains susceptible to most prescribed antibiotics, a significant number of treatment failures have been reported (Trieu-Cuot et al., 1993; Arthur et al., 1995; González Pedraza Avilés and Ortiz Zaragoza, 2000; Schwarz et al., 2004; Woodford, 2005; Pletz et al., 2006; Kimura et al., 2013; Cattoir, 2016; Doumith et al., 2017; Lai et al., 2017). Although tetracycline is not indicated for bovine mastitis-associated streptococci treatment in Portugal nowadays, high resistance to this class of antibiotics has been observed among bovine SDSD isolates. We observed a significant increase in the frequency of *tetM*, *tetK*, and *tetO* tetracycline resistance genes among bovine SDSD of collection II compared with bovine strains of the collection I. This frequency is likely associated with the fact that tetracycline has been extensively used to treat infections in bovine for many decades; tetracycline resistance determinants are linked to other resistance genes. For example, tetracycline resistance genes can be acquired via MGEs (e.g., plasmids and/or transposons), which also harbored erythromycin resistance genes (Brenciani et al., 2007, 2010; Emaneini et al., 2014; Santoro et al., 2014; Cattoir, 2016), contributing to a multiresistant phenotype. In our SDSD isolates, we identified the presence of *ermB*/*tetO*/*tetK* and *ermB*/*tetO* macrolide/tetracycline resistance genes, suggesting a possible horizontal gene co-transference of these genes.

Since the sequential order of the spacers in the CRISPR array provides chronological information about the bacteria’s exposure to foreign nucleic acids (Lier et al., 2015), providing insights into host–phage interactions within a specific niche, herein, we investigated the occurrence of CRISPR/Cas systems and CRISPR spacer content in bovine SDSD isolates to gain insight into the population diversity and the impact of this system on HGT.

Our results showed that all bovine SDSD strains characterized in the present study carried the CRISPR/Cas IIA system. In addition, we also observed a high degree of polymorphism in the CRISPR spacers providing a high ability to discriminate between strains. About 18.4% of spacers match known prophages with over 96% identity indicating a cross-species exchange of genetic material. The most frequent bacteriophages are listed in Table 2. Some studies have suggested that some common phages act as spacer donators (Le Rhun et al., 2019; Hu et al., 2020); thus, different spacers in different strains but identified as the same phage may be correlated with phage’s evolution (Sorek et al., 2013; Landsberger et al., 2018; Watson et al., 2018). About 28% of the sequence spacers of SDSD were not identified in the database, probably because of the rapid evolution of phage sequences, and sequences of new phages are not available.

The widespread presence of this CRISPR/Cas system among SDSD bovine strains, the high conservation of repeated sequences, and the polymorphism observed among the CRISPR IIA array spacers of these strains may lead us to consider them as indicators of the activity of the system (Figure 5 and Supplementary Table 9 in Supplementary File 6).

To elucidate the relationship between the prophages and CRISPR systems among SDSD strains, we analyzed CRISPR array, CRISPR-associated (Cas) proteins, and prophage structure using complete genome sequences of SDSD listed in Supplementary Table 1 (Supplementary File 1). For a comparative analysis, complete genome of SDSE, *S. canis*, and *S. pyogenes* were also analyzed.

We observed that the CRISPR II-A system had increased the number of spacers relative to CRISPR I-C. Indeed, CRISPR II-A locus is the most widespread type among streptococci strains and has the most significant number of spacers. Several studies concluded that type II-A CRISPR might be the primary phage prevention or elimination system in the streptococcus genome (Serbanescu et al., 2015; Yamada et al., 2019; Hu et al., 2020).

The genomes of SDSD and SDSE show a high number of spacers compared with those of *S. pyogenes* (Table 3). Some studies have speculated on the loss of the CRISPR array and/or the CRISPR/Cas locus in some clinical isolates. In this regard, it was proposed that the lack of CRISPR/Cas could indicate an adaptation to acquire new virulence genes. It has also been hypothesized that the low number of spacers acquired by *S. pyogenes* could be a strategy to acquire virulence genes and thus increase its pathogenic capacity (Le Rhun et al., 2019). Among our SDSD strains, there was no statistically significant difference in the total number of prophages in the strains with type IC and type IIA systems. A more significant number of prophage regions would be expected in the absence of the CRISPR/Cas system; however, we observe SDSD strains with an active CRISPR/Cas system harboring several prophage regions per genome.

Despite the different origins of SDSD strains, most prophages are related and were identified initially in *S. pyogenes* (Table 4). The phages’ specificity can explain the high frequency of *S. pyogenes* prophages among SDSD isolates; that is, the interaction between phage particles and bacteria generally involves specific receptors located in the bacterial cell membranes (Stone et al., 2019). The fact that SDSD and *S. pyogenes* do not share the same host makes it seem likely that the transfer of phages harboring streptococcal pyrogenic exotoxins has occurred before the divergence of ecological niches between the two species.

In conclusion, our results showed a significant increase in the frequencies of the *tetM*, *tetO*, and *tetK* genes over time, although tetracycline is not indicated for bovine mastitis-associated streptococcus treatment in Portugal nowadays. Our data suggest that carriage of *S. pyogenes* prophage virulence genes (*speC*, *speK*, *speL*, *speM*, and *spd1*) is maintained over time and seems to be a property of bovine SDSD, but not of human and fish SDSD and SDSE strains. The high homology of SAgs genes between bovine SDSD and *S. pyogenes* strains may suggest that the HGT events occurred before habitat separation.

This study documented for the first time the widespread presence of the CRISPR/Cas IIA system among bovine SDSD isolates, and the polymorphism observed in spacer content can be considered indicators of the system activity. However, no correlation was observed between the number of spacers CRISPR IIA and the number of prophages in the SDSD genomes. Further studies are necessary to understand better the CRISPR/Cas systems among SDSD strains, such as other CRISPR systems, and its impact on the evolution of SDSD virulence.

Our data are the first to suggest that hemolytic differences between human SDSD strains (β-hemolytic) and bovine SDSD isolates (α-hemolytic) may be related to the loss of SagB-I and mutation of *sagA* gene observed in bovine SDSD isolates. The SagA alignment reveals higher homology between human and fish SDSD and SDSE strains when compared with the bovine SDSD isolates. The close genetic relationship between non-bovine SDSD and SDSE was also clear from phylogenetic analysis based on MLSA, while bovine SDSD isolates seem more divergent.

The data set of this study suggests that the separation between the subspecies “*dysgalactiae*” and “*equisimilis*” should be reconsidered. However, a study including the most comprehensive collection of strains from different environments would be required for definitive conclusions regarding the two taxa.

## Data Availability Statement

The datasets presented in this study can be found in online repositories. The names of the repository/repositories and accession number(s) can be found in the article/Supplementary Material.

## Author Contributions

IS-S, CA-B, AF, and RM contributed to the idea or design of the research. RB and MO collaborated with the collection of bovine mastitis samples. CA-B contributed to the experimental work, analysis of sequences, and performed complete genomes. JC and CR-R contributed to the research of virulence genes. LC and RT contributed with the support in the BioNumerics software and assistance in the analysis. CA-B wrote down the first draft and the subsequent revisions of the manuscript. RM and AF performed the revision and editing of the final manuscript. All authors contributed to the article and approved the submitted version.

## Conflict of Interest

The authors declare that the research was conducted in the absence of any commercial or financial relationships that could be construed as a potential conflict of interest.

## Footnotes

1 www.ncbi.nlm.nih.gov/BLAST

2 https://crisprcas.i2bc.paris-saclay.fr/CrisprCasFinder/Index

3 http://www.microbiome-bigdata.com/CRISPRminer/index.php/Home/Index/inde
