# Supplementary material for: New Insights on Streptococcus dysgalactiae subsp. dysgalactiae Isolates
Source: Front Microbiol. 2021 Jul 15;12:686413. doi: 10.3389/fmicb.2021.686413 (PMC8319831; doi:10.3389/fmicb.2021.686413)
Supplement: Supplementary file 1 [file Data_Sheet_1.PDF]

S1 Table. *Streptococcus* genomes used in the study.

STRAIN	ACCESSION NUMBER	TOTAL LENGTH (MB)	PROTEIN COUNT	GC%	Host
**SDSD ATCC 27957**	NZ_CM001076.1	2.14184	3972	39.4	Bovine
**SDSD DB31752-13**	NZ_CP033164.1	2.31957	2026	39.3	Human
**SDSD DB49998-05**	NZ_CP033163.1	2.17141	1850	39.4	Human
**SDSD DB60705-15**	NZ_CP033165.1	2.19066	1944	39.4	Human
**SDSD DB53993-17**	INSDC: REFX00000000.1	2.17042	1966	39.6	Human
**SDSD Kdys0611**	NZ_ AP018726.1	2.22482	1961	39.9	Fish
**SDSD NCTC13731**	NZ_UHFH00000000.1	2.1517	1920	39.2	Bovine
**SDSD NCTC4669**	NZ_LR134094.1	2.20189	1987	39.2	Bovine
**SDSD NCTC4670**	NZ_UHFG00000000.1	2.25218	2032	39.3	Bovine
**SDSD STREP97-15**	NZ_CP033166.1	2.43257	2211	39.5	Fish
**SDSE NCTC11554**	NZ_LR594047.1	2.23328	2062	39.5	Human
**SDSE NCTC5370**	NZ_LS483318.1	2.07995	1898	39.5	not available
**SDSE NCTC5371**	NZ_LS483367.1	2.10376	1858	39.6	not available
**SDSE NCTC6179**	NZ_LS483361.1	2.2552	2033	39.8	Horse
**SDSE NCTC6181**	NZ_LR134316.1	2.2699	2049	39.7	not available
**SDSE NCTC7136**	NZ_LS483413.1	2.24923	2084	39.4	not available
**SDSE NCTC9413**	NZ_LR594045.1	2.24174	2019	39.5	Human
**SDSE NCTC9414**	NZ_LS483363.1	2.16967	2026	39.5	Human
**SDSE 167**	NZ_AP012976.1	2.0764	1647	39.6	Human
**SDSE AC-2713**	NZ_HE858529.1	2.17944	1941	39.5	Human
**SDSE ATCC 12394**	NZ_CP002215.1	2.15949	1893	39.5	Human
**SDSE GGS 124**	NZ_AP010935.1	2.10634	1931	39.6	Human
**SDSE RE378**	NZ_AP011114.1	2.15115	1866	39.5	Human
**SD NCTC6403**	NZ_LR594046.1	2.2005	1965	39.5	Not available
**SD NCTC13759**	INSDC: UHFJ00000000.1	2.15094	1929	38.3	Not available
**SD FDAARGOS 654**	NZ_CP044102.1	2.25353	2045	39.1	Human
**SD SCDR-SD1**	NZ_CP033391.1	2.17914	1725	41	Human
***S. canis* B700072**	NZ_LR590625.1	2.10661	1867	39.7	Not available
***S. canis* NCTC12191**	NZ_LR134293.1	2.08474	1855	39.9	Not available
***S. canis* HL_100**	NZ_CP046521.1	2.17824	1952	39.7	Dog
***S. pyogenes* emm64.3**	NZ_CP035435.1	1.77977	1693	38.6	Human
***S. pyogenes* emm70**	NZ_CP035448.1	1.82647	1707	38.5	Human
***S. pyogenes* emm97.1**	NZ_CP035447.1	1.81209	1677	38.4	Human
***S. pyogenes* FDAARGOS_668**	NZ_CP044093.1	1.84135	1737	38.4	Human
***S. pyogenes* HKU360**	NZ_CP009612.1	1.94454	1832	38.5	Human
***S. pyogenes* JS12**	NZ_CP021640.1	1.81028	1632	38.5	Human
***S. pyogenes* NCTC10085**	NZ_LS483401.1	1.79527	1679	38.6	Human
***S. pyogenes* NCTC12052**	NZ_LS483352.1	1.85773	1791	38.5	Human
***S. pyogenes* NCTC12696**	NZ_LS483332.1	1.84505	1777	38.5	Human
***S. pyogenes* NCTC13736**	NZ_LS483414.1	1.91321	1814	38.6	Human
***S. pyogenes* NCTC13738**	NZ_LS483382.1	1.84734	1746	38.6	Human
***S. pyogenes* NCTC8322**	NZ_LS483520.1	1.82959	1746	38.5	Human
***S. pyogenes* SP1336**	NZ_CP031738.1	1.87883	1736	38.5	Human
