# Supplementary material for: New Insights on Streptococcus dysgalactiae subsp. dysgalactiae Isolates
Source: Front Microbiol. 2021 Jul 15;12:686413. doi: 10.3389/fmicb.2021.686413 (PMC8319831; doi:10.3389/fmicb.2021.686413)
Supplement: Supplementary file 2 [file Data_Sheet_2.PDF]

S2 Table: Primer sequences, amplicon expected sizes PCR conditions for amplification of all the genetic determinants.

Primer name	Sequence (5'-3')	Product	Reference
<u>**Pyrogenic exotoxins:**</u>			
**speA (for.)**	ATGGAAAACAATAAAAAAGTATTG	755	Matsumoto et al., 2003
**speA (rev.)**	TTACTTGGTTGTTAGGTAGACTTC		
**speB (for.)**	TTCTAGGATACTCTACCAGC	300	Jasir *et al*., 2001
**speB (rev.)**	ATTTGAGCAGTTGCAGTAGC		
**speC (for.)**	GCAGGGTAAATTTTTCAACGACACACA	407	Rato *et al.*, 2011
**speC (rev.)**	TGTGCCAATTTCGATTCTGCCGC		
**speF (for.)**	TACTTGGATCAAGACG	782	Schmitz et al., 2003
**speF (rev.)**	GTAATTAATGGTGTAGCC		
**speG (for.)**	TGTATCTTTAGGGATTACTGATCAG	389	Rato et al., 2011
**speG (rev.)**	CTCGACCTAAAAGCTTATCATCCTT		
**speH (for.)**	AGATTGGATATCACAGG	416	Pires *et al*., 2009
**speH (rev.)**	CTATTCTCTCGTTATTGG		
**speK (for.)**	TACAAATGATGTTAGAAATCCAAGGAACATATATGCT	656	Rato *et al.*, 2011
**speK (rev.)**	CAAAGTGACTTACTTTACTCATATCAATCGTTTC		
**speL (for.)**	CTGTTAGGATGGTTTCTGCGGAAGAG	605	Rato *et al.*, 2011
**speL (rev.)**	AGCACCTTCCTCTTTCTCGCCT		
**speM (for.)**	CCAATATGAAGATAACAAAGAAAATTGGCACCC	600	Rato *et al.*, 2011
**speM (rev.)**	CAAAGTGACTTACTTTACTCATATCAATCG		
**spegg (for.)**	GCTTATGATGTTACTCCACTTGA	420	Rato et al., 2011
**spegg (rev.)**	ATAACGCGATTCCGAATCATAGA		
<u>**Mitogenic exotoxin Z:**</u>			
**smeZ (for.)**	CAGATATAGTAATTGATTTTA	399	Darenberg *et al.*, 2007
**smeZ (rev.)**	AGCTAGAACCAGAAGAATAT		
<u>**DNAse1:**</u>			
**spd1 (for.)**	CCCTTCAGGATTGCTGTCAT	400	Green *et al*., 2005
**spd1 (rev.)**	ACTGTTGACGCAGCTAGGG		
<u>**Streptodornase**</u>			
**sdn (for.)**	ACCCCATCGGAAGATAAAGC	489	Matsumoto *et al*., 2003
**sdn (rev.)**	AACGTTCAACAGGCGCTTAC		
<u>**Streptolysin S**</u>			
**sagA (for.)**	TACTTCAAATATTTTAGCTACT	487	Abdelsalam *et al*., 2010
**sagA (rev.)**	GATGATACCCCGATAAGGATAA		
**sagB (for.)**	ACAATCGTCCCCCCCTAA	519	
**sagB (rev.)**	GGAGAGTAATCGGGTATA		
**sagE (for.)**	TGGGAAAGGAAGTAGTGG	304	
**sagE (rev.)**	AGCTAGAAGCAAAGGATAGA		This study
**sagH (for.)**	GCAACAACACACAACGAA	411	
**sagH (rev.)**	TGCCCACCAAAAATAAGG		
**sagI (for.)**	GCTCAGCTAAAACAAACGAA	763	
**sagI (rev.)**	AAGAAGGGGTAATGGACAA		
<u>**Macrolide Resistance**</u>			
**mefA (for)**	GACCAAAAGCCACAATTGTGGA	1432	Pires *et al*., 2005
**mefA (rev.)**	CCTCCTGTCTATAATCGCATG		
**ermA (for.)**	CCCGAAAAATACGCAAAATTTCAT	590	Pires *et al*., 2005
**ermA (rev.)**	CCCTGTTTACCCATTTATAAACG		
**ermB (for.)**	GGAGTGATACATGAACAAAAATA	531	Pires *et al*., 2005
**ermB (rev.)**	TTCCTTTTAGTAACGTGTAACTTT		
<u>**Tetracycline Resistance**</u>			
**tetM (for.)**	TGGAATTGATTTATCAACGG	1080	Pires *et al*., 2005
**tetM (rev.)**	TTCCAACCATACAATCCTTG		
**tetO (for.)**	AACTTAGGCATTCTGGCTCAC	515	Ng *et al.*, 2001
**tetO (rev.)**	TCCCACTGTTCCATATCGTCA		
**tetT (for.)**	AAGGTTTATTATATAAAAGTG	169	Aminov *et al*., 2001
**tetT (rev.)**	AGGTGTATCTATGATATTTAC		
**tetW (for.)**	GAGAGCCTGCTATATGCCAGC	168	Aminov *et al*., 2001
**tetW (rev.)**	GGGCGTATCCACAATGTTAAC		
**tetQ (for.)**	TTATACTTCCTCCGGCATCG	904	Ng *et al.*, 2001
**tetQ (rev.)**	ATCGGTTCGAGAATGTCCAC		
**tetS (for.)**	GAAAGCTTACTATACAGTAGC	169	Aminov *et al*., 2001
**tetS (rev.)**	AGGAGTATCTACAATATTTAC		
**tetL (for.)**	TCGTTAGCGTGCTGTCATTC	267	Ng *et al.*, 2001
**tetL (rev.)**	GTATCCCACCAATGTAGCCG		

**tetK (for.)**	TCGATAGGAACAGCAGTA	169	Ng *et al.*, 2001
**tetK (rev.)**	CAGCAGATCCTACTCCTT		
**Lincosamide Resistance**			
**linB (for.)**	CCTACCTATTGTTTGTGGAA	925	Bozdogan *et al*., 1999
**linB (rev.)**	ATAACGTTACTCTCCTATTC		
**Type II CRISPR/Cas**			
**cas9 (for.)**	GAAATACAGACCGCCACA	873	
**cas9 (rev.)**	CATCGTAGCGCTTAATCA		
**cas1 (for.)**	ACCCACTCGAAATTATCCT	557	
**cas1 (rev.)**	CCAGACACAACCACTTCA		
**cas4 (for.)**	CTTTTGGTCTCACGTGCT	435	This study
**cas4 (rev.)**	TTTTTTACTCAACCTGGGCT		
**csn2 (for.)**	TAAGAGGCGGTACAATTC	499	
**csn2 (rev.)**	CTAGCCACTTCATCCTTT		
**CRISPR Sdys (for.)**	TAGAAAGAATGGGAAGGCA	variable	
**CRISPR Sdys (rev.)**	AATACAGGGGCTTTTCAAGA		
