# Supplementary material for: New Insights on Streptococcus dysgalactiae subsp. dysgalactiae Isolates
Source: Front Microbiol. 2021 Jul 15;12:686413. doi: 10.3389/fmicb.2021.686413 (PMC8319831; doi:10.3389/fmicb.2021.686413)
Supplement: Supplementary file 3 [file Data_Sheet_3.PDF]

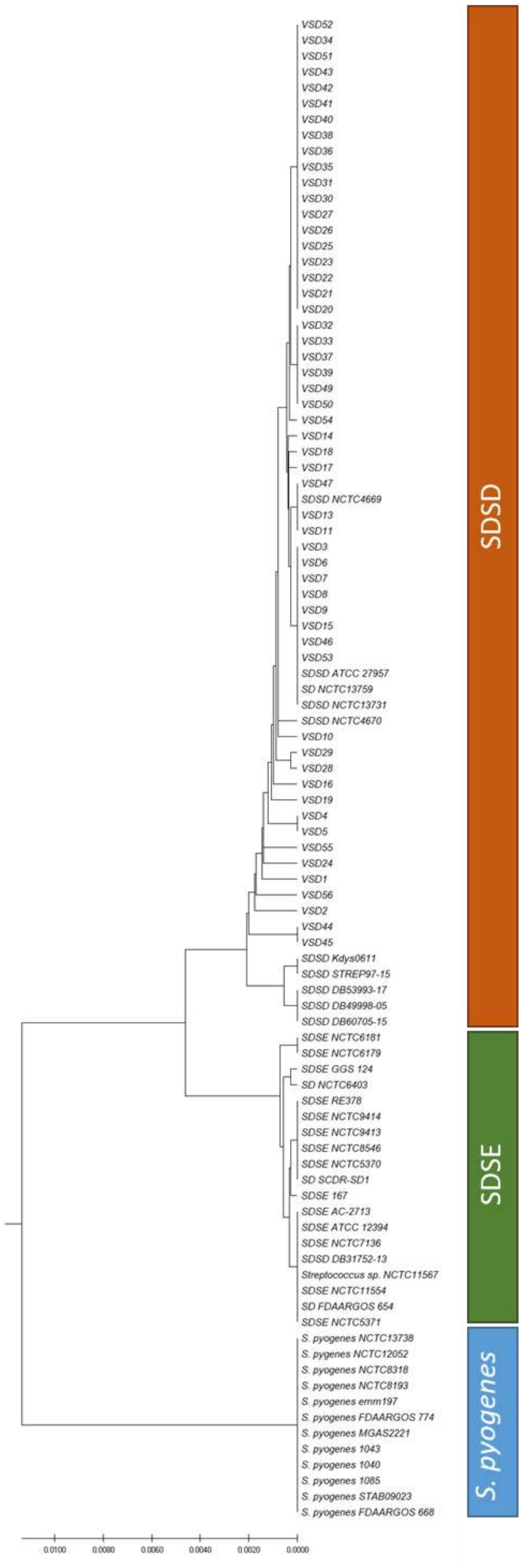

S1 Figure. UPGMA dendrogram generated by multiple alignment of rRNA 16S sequence of: i) bovine *Streptococcus dysgalactiae* subspecies *dysgalactiae* isolates (SDSD, n= 55), VSD1 to VSD11 and VSD13 to VSD19 from collection I (2002-2003) and VSD20 to VSD56 from collection II (2011-2013); ii) SDSD sequences deposited in the GenBank database (n= 12); iii) *Streptococcus dysgalactiae* subspecies *equisimilis* isolates (SDSE, n= 16) and iv) *Streptococcus pyogenes* (*S. pyogenes*, n= 12) available at the National Centre for Biotechnology Information. GenBank accession numbers is shown in Table 1. The DNA sequence was analyzed by CLC Bio Main Workbench 20.1 alignment program editor (QIAGEN, Netherlands). The optimal tree with the sum of branch length = 0.06766642 is shown. The tree is drawn to scale, with branch lengths in the same units as those of the evolutionary distances used to infer the phylogenetic tree. The evolutionary distances were computed using the Kimura 2-parameter method and are in the units of the number of base substitutions per site. This analysis involved 94 nucleotide sequences. All ambiguous positions were removed for each sequence pair (pairwise deletion option). There were a total of 1432 positions in the final dataset. Evolutionary analyses were conducted in MEGA X software (Kumar et al. 2018). Sequence analysis of the rRNA 16S from bovine SDSD strains showed between 99.2% and 100 % nucleotide identity to SDSD ATCC 2795, SDSD NCTC4670, SDSD NCTC13731 and SDSD NCTC4669 strains deposited in the National Center for Biotechnology Information Nucleotide database.

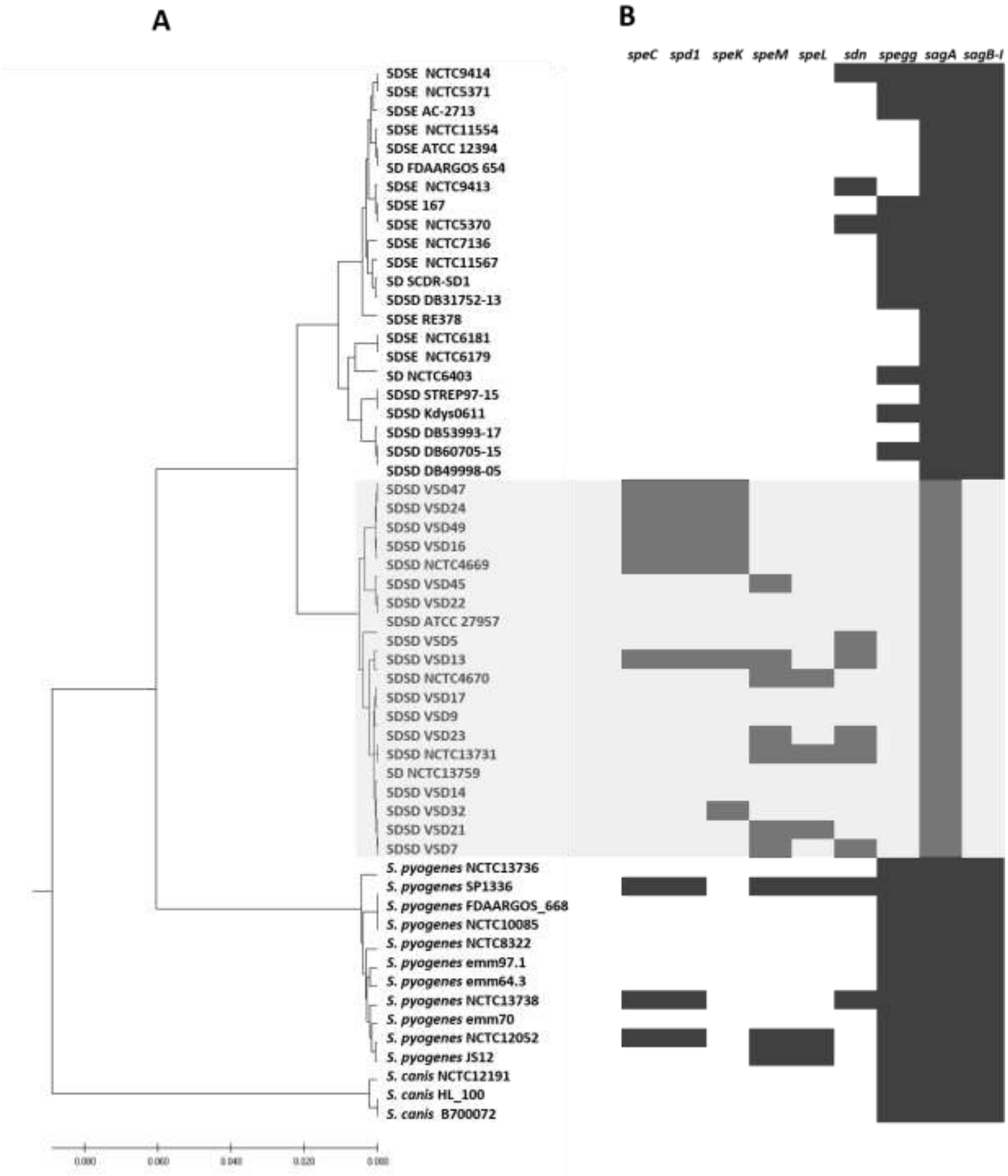

S2 Figure. Molecular marker hierarchical clustering dendrogram compared to virulence genes profiles. (A) Hierarchical clustering dendrogram generated by multiple alignment of concatenated sequences of seven housekeeping genes (*gki, gtr, murI, mutS, recP, xpt,* and *atoB*). Cluster analysis of similarity matrices was calculated with the unweighted pairgroup method with arithmetic means. The distances were computed using the Kimura 2-parameter method and are in the units of the number of base substitutions per site. All ambiguous positions were removed for each sequence pair (pairwise deletion option). Evolutionary analyses were conducted in MEGA X. (B) Graphic representation of the virulence gene profile. *S. pyogenes* virulence genes encoding phage: *speC, speK, speL, speM, spegg* - streptococcal pyrogenic exotoxins; *spd1* - DNase*; sdn* streptodornase. *sagA* to *sagI* – Streptolysin (SLS) operon is organized into nine genes *sagA* to *sagI*. SagBCD is responsible for the conversion of SagA into SLS. The role of the remaining Sag proteins is not yet clear. SagF is membrane-associated and SagE is a peptidase responsible for leader cleavage. SagG, SagH, and SagI are thought to be membrane proteins that form an (ABC)-type transporter.
