# Supplementary material for: New Insights on Streptococcus dysgalactiae subsp. dysgalactiae Isolates
Source: Front Microbiol. 2021 Jul 15;12:686413. doi: 10.3389/fmicb.2021.686413 (PMC8319831; doi:10.3389/fmicb.2021.686413)
Supplement: Supplementary file 4 [file Data_Sheet_4.PDF]

Table S4. Nucleotide sequence identity (%) obtained from multiple alignment of ***speC-spd1*** sequences the sequences of the streptococcal species deposited in the GenBank database using BlastN (http://www.ncbi.nlm.nih.gov/BLAST/).

ID	DESCRIPTION	%IDENTITY	E-VALUE	SCORE	HSP START	HSP END	OVERLAP
LR134094.1	SDSD strain NCTC4669*	100,00	0,00	2 912,00	520398	521979	100,00
CP044093.1	*S. pyogenes* strain FDAARGOS_668	99,18	0,00	2 847,00	238495	236914	99,87
LR590466.1	*S. pyogenes* strain NCTC8193	99,18	0,00	2 847,00	1271944	1270363	99,87
CP033907.1	*S. pyogenes* strain Duke-Large	99,18	0,00	2 847,00	588697	590278	100,00
CP033908.1	*S. pyogenes* strain RLGH	99,18	0,00	2 847,00	588676	590257	100,00
CP033767.1	*S. pyogenes* strain FDAARGOS_534	99,18	0,00	2 847,00	597016	598597	100,00
CP031738.1	*S. pyogenes* strain SP1336	99,18	0,00	2 847,00	571005	572586	100,00
LS483437.1	*S. pyogenes* strain NCTC13751	99,18	0,00	2 847,00	567242	568823	100,00
LS483391.1	*S. pyogenes* strain NCTC8320	99,18	0,00	2 847,00	578006	579587	100,00
LS483357.1	*S. pyogenes* strain NCTC8326	99,18	0,00	2 847,00	1291590	1290009	99,87
LS483351.1	*S. pyogenes* strain NCTC8195	99,18	0,00	2 847,00	630633	632214	100,00
CP020082.1	*S. pyogenes* strain STAB120304	99,18	0,00	2 847,00	567602	569183	100,00
CP014542.1	*S. pyogenes* strain STAB14018	99,18	0,00	2 847,00	566303	567884	100,00
CP014138.1	*S. pyogenes* strain MEW427	99,18	0,00	2 847,00	549849	551430	100,00
CP007560.1	*S. pyogenes* strain NGAS743	99,18	0,00	2 847,00	1255598	1254017	99,87
CP008695.1	*S. pyogenes* strain M23ND	99,18	0,00	2 847,00	207958	209539	100,00
AE009949.1	*S. pyogenes* MGAS8232	99,18	0,00	2 847,00	617201	618782	100,00
CP000260.1	*S. pyogenes* MGAS10270	99,18	0,00	2 847,00	570745	572326	100,00
CP000259.1	*S. pyogenes* MGAS9429	99,18	0,00	2 847,00	569477	571058	100,00
CP029694.1	*S. pyogenes* strain ABC020055975	99,12	0,00	2 842,00	1314809	1313228	99,87
CP000262.1	*S. pyogenes* MGAS10750	99,12	0,00	2 842,00	587923	589504	100,00
CP035455.1	*S. pyogenes* strain emm197	99,05	0,00	2 837,00	686904	688485	100,00
CP000056.2	*S. pyogenes* MGAS6180	99,05	0,00	2 837,00	988406	986825	99,87
CP031617.1	*S. pyogenes* strain MGAS29409	99,05	0,00	2 837,00	887725	889306	100,00
CP031618.1	*S. pyogenes* strain MGAS29326	99,05	0,00	2 837,00	900693	902274	100,00
CP031619.1	*S. pyogenes* strain MGAS29284	99,05	0,00	2 837,00	930948	932529	100,00
CP031620.1	*S. pyogenes* strain MGAS29064	99,05	0,00	2 837,00	847632	849213	100,00
CP031621.1	*S. pyogenes* strain MGAS28746	99,05	0,00	2 837,00	847195	848776	100,00
CP031622.1	*S. pyogenes* strain MGAS28686	99,05	0,00	2 837,00	888771	890352	100,00
CP031623.1	*S. pyogenes* strain MGAS28669	99,05	0,00	2 837,00	858620	860201	100,00
CP031624.1	*S. pyogenes* strain MGAS28650	99,05	0,00	2 837,00	845757	847338	100,00
CP031625.1	*S. pyogenes* strain MGAS28533	99,05	0,00	2 837,00	887312	888893	100,00
CP031626.1	*S. pyogenes* strain MGAS28386	99,05	0,00	2 837,00	845804	847385	100,00
CP031627.1	*S. pyogenes* strain MGAS28360	99,05	0,00	2 837,00	846064	847645	100,00
CP031629.1	*S. pyogenes* strain MGAS28278	99,05	0,00	2 837,00	1028846	1027265	99,87
CP031631.1	*S. pyogenes* strain MGAS28191	99,05	0,00	2 837,00	846016	847597	100,00
CP031632.1	*S. pyogenes* strain MGAS28078	99,05	0,00	2 837,00	846714	848295	100,00
CP031632.1	*S. pyogenes* strain MGAS28078	99,05	0,00	2 837,00	1307202	1305621	99,87
CP031633.1	*S. pyogenes* strain MGAS11115	99,05	0,00	2 837,00	844908	846489	100,00
CP031634.1	*S. pyogenes* strain MGAS11108	99,05	0,00	2 837,00	847486	849067	100,00
CP031635.1	*S. pyogenes* strain MGAS11052	99,05	0,00	2 837,00	901853	903434	100,00
CP031636.1	*S. pyogenes* strain MGAS10826	99,05	0,00	2 837,00	845847	847428	100,00
CP031637.1	*S. pyogenes* strain MGAS10786	99,05	0,00	2 837,00	888553	890134	100,00
CP031639.1	*S. pyogenes* strain MGAS7914	99,05	0,00	2 837,00	846674	848255	100,00
CP032700.1	*S. pyogenes* strain TSPY556	99,05	0,00	2 837,00	663278	664859	100,00
CP033335.1	*S. pyogenes* strain TSPY208	99,05	0,00	2 837,00	1176039	1174458	99,87
LR134314.1	*S. pyogenes* strain NCTC8302	99,05	0,00	2 837,00	574258	572677	99,87
LS483329.1	*S. pyogenes* strain NCTC12058	99,05	0,00	2 837,00	514091	515672	100,00
LS483394.1	*S. pyogenes* strain NCTC10880	99,05	0,00	2 837,00	1109755	1108174	99,87
LS483386.1	*S. pyogenes* strain NCTC13742	99,05	0,00	2 837,00	1023500	1021919	99,87

*SDSD strain NCTC4669 ***speC-spd1*** sequences was used as reference for alignment

Table S5. Nucleotide sequence identity (%) obtained from multiple alignment of ***speK*** sequences the sequences of the streptococcal species deposited in the GenBank database using BlastN.

ID	DESCRIPTION	%IDENTITY	E-VALUE	BIT SCORE	HSP START	HSP END	OVERLAP
SDSD_NCTC4669	SDSD strain NCTC4669*	100	0,00	1 160,85	519541	520188	100,00
HQ724300.1	SDSD speK-1 allele	99,81	0,00	932,73	130	648	100,00
HQ724302.1	SDSD speK-3 allele	99,61	0,00	928,22	130	648	100,00
HQ724301.1	SDSD speK-2 allele	99,61	0,00	928,22	130	648	100,00
CP035449.1	*S. pyogenes* strain emm56	99,42	0,00	923,71	656287	656805	100,00
CP035435.1	*S. pyogenes* strain emm64.3	99,42	0,00	923,71	665285	665803	100,00
MK448977.1	S. phage Javan530	99,42	0,00	923,71	39373	39891	100,00
MK448962.1	S. phage Javan496	99,42	0,00	923,71	30739	31257	100,00
MK448950.1	S. phage Javan464	99,42	0,00	923,71	40097	40615	100,00
MK448864.1	S. phage Javan180	99,42	0,00	923,71	28860	29378	100,00
MK448791.1	S. phage Javan517	99,42	0,00	923,71	39892	40410	100,00
MK448788.1	S. phage Javan511	99,42	0,00	923,71	39373	39891	100,00
CP033815.1	*S. pyogenes* strain FDAARGOS_514	99,42	0,00	923,71	270982	270464	99,61
CP000056.2	*S. pyogenes* MGAS6180	99,42	0,00	923,71	1229272	1228754	99,61
CP031622.1	*S. pyogenes* strain MGAS28686	99,42	0,00	923,71	647904	648422	100,00
CP031625.1	*S. pyogenes* strain MGAS28533	99,42	0,00	923,71	646445	646963	100,00
CP031640.1	*S. pyogenes* strain MGAS7888	99,42	0,00	923,71	1254573	1254055	99,61
LS483437.1	*S. pyogenes* strain NCTC13751	99,42	0,00	923,71	1286072	1285554	99,61
LS483437.1	*S. pyogenes* strain NCTC13751	72,64	2,22E-74	291,63	1185047	1184535	98,46
LS483432.1	*S. pyogenes* strain NCTC13745	99,42	0,00	923,71	1232381	1231863	99,61
LS483421.1	*S. pyogenes* strain NCTC10877	99,42	0,00	923,71	673253	673771	100,00
AP018337.1	*S. pyogenes* DNA, strain: KS030	99,42	0,00	923,71	670525	671043	100,00
CP020082.1	*S. pyogenes* strain STAB120304	99,42	0,00	923,71	1285904	1285386	99,61
CP020082.1	*S. pyogenes* strain STAB120304	72,64	2,22E-74	291,63	1184879	1184367	98,46
CP022206.1	*S. pyogenes* strain GURSA1	99,42	0,00	923,71	727035	727553	100,00
CP022354.1	*S. pyogenes* strain GUR	99,42	0,00	923,71	727035	727553	100,00
CP021972.1	S. equi subsp. equi ATCC 39506	99,42	0,00	923,71	1788081	1787563	99,61
CP014542.1	*S. pyogenes* strain STAB14018	99,42	0,00	923,71	1285757	1285239	99,61
CP014542.1	*S. pyogenes* strain STAB14018	72,64	2,22E-74	291,63	1184732	1184220	98,46
CP011535.2	*S. pyogenes* strain M28PF1	99,42	0,00	923,71	649318	649836	100,00
CP013672.1	*S. pyogenes* strain AP53	99,42	0,00	923,71	1006304	1005786	99,61
AP014596.1	*S. pyogenes* DNA, strain: M3-b	99,42	0,00	923,71	664401	664919	100,00
CP010450.1	*S. pyogenes* strain NGAS638	99,42	0,00	923,71	960926	960408	99,61
CP011068.1	*S. pyogenes* strain STAB10015	99,42	0,00	923,71	1282952	1282434	99,61
CP007562.1	*S. pyogenes* strain NGAS327	99,42	0,00	923,71	759236	759754	100,00
KM872099.1	*S. pyogenes* strain P19 SpeL gene	99,42	0,00	923,71	102	620	100,00
CP007041.1	*S. pyogenes* STAB902	99,42	0,00	923,71	664565	665083	100,00
CP003121.1	*S. pyogenes* MGAS1882	99,42	0,00	923,71	1101251	1100733	99,61
FM204883.1	S. equi subsp. equi 4047	99,42	0,00	923,71	1743964	1743446	99,61
CP000260.1	*S. pyogenes* MGAS10270	99,42	0,00	923,71	1256821	1256303	99,61
CP000003.1	*S. pyogenes* MGAS10394	99,42	0,00	923,71	989602	989084	99,61
BA000034.2	*S. pyogenes* SSI-1 DNA	99,42	0,00	923,71	664442	664960	100,00
AE014074.1	*S. pyogenes* MGAS315	99,42	0,00	923,71	1232443	1231925	99,61
CP035427.1	*S. pyogenes* strain emm74	99,23	0,00	919,20	1189473	1188955	99,61
LR134389.1	S. equi subsp. equi strain NCTC9682	99,23	0,00	919,20	1745138	1744620	99,61

*SDSD strain NCTC4669 ***speK*** sequence was used as reference for alignment

Table S6. Nucleotide sequence identity (%) obtained from multiple alignment of ***speL*** sequences the sequences of the streptococcal species deposited in the GenBank database using BlastN.

ID	DESCRIPTION	%IDENTITY	E-VALUE	HSP START	HSP END	OVERLAP	%GAPS
LR134094.1	SDSD strain NCTC4669	100,00	0,00	1862333	1861864	99,57	0,00
UHFH00000000.1	SDSD NCTC13731	100,00	0,00	530999	531468	100,00	0,00
UHFG00000000.1	SDSD NCTC4670	97,45	0,00	1500252	1499783	99,57	0,00
HQ724303.1	SDSD pyrogenic exotoxin L*	100,00	0,00	1	470	100,00	0,00
LR134389.1	*S. equi* subsp. *equi* strain NCTC9682	99,57	0,00	1744176	1743707	99,57	0,00
CP021972.1	S. equi subsp. equi strain ATCC39506	99,57	0,00	1787119	1786650	99,57	0,00
FM204883.1	S. equi subsp. equi 4047	99,57	0,00	1743002	1742533	99,57	0,00
AE009949.1	*S. pyogenes* MGAS8232	97,66	0,00	1042269	1041800	99,57	0,00
CP035455.1	*S. pyogenes* strain emm197	97,65	0,00	848404	848872	99,79	0,00
CP035454.1	*S. pyogenes* strain emm54	97,65	0,00	967385	966917	99,36	0,00
CP035453.1	*S. pyogenes* strain emm100	97,65	0,00	1018376	1017908	99,36	0,00
CP035436.1	*S. pyogenes* strain emm89.14	97,65	0,00	802717	803185	99,79	0,00
CP007023.1	*S. pyogenes* STAB1102 genome	97,65	0,00	940640	940172	99,36	0,00
CP006366.1	*S. pyogenes* HSC5	97,65	0,00	965169	964701	99,36	0,00
CP035432.1	*S. pyogenes* strain emm11	97,45	0,00	1153816	1153347	99,57	0,00
CP031628.1	*S. pyogenes* strain MGAS28330	97,45	0,00	846146	846615	100,00	0,00
CP033621.1	*S. pyogenes* strain M75	97,45	0,00	1145509	1145040	99,57	0,00
CP033335.1	*S. pyogenes* strain TSPY208	97,45	0,00	982160	981691	99,57	0,00
LR134272.1	*S. pyogenes* strain NCTC12060	97,45	0,00	952627	952158	99,57	0,00
CP021640.1	*S. pyogenes* strain JS12	97,45	0,00	975132	974663	99,57	0,00
CP020082.1	*S. pyogenes* strain STAB120304	97,45	0,00	1183908	1183439	99,57	0,00
CP020027.1	*S. pyogenes* strain STAB090229	97,45	0,00	1139634	1139165	99,57	0,00
CP014542.1	*S. pyogenes* strain STAB14018	97,45	0,00	1183761	1183292	99,57	0,00
AF514283.1	*S. pyogenes* speM gene, partial cds	97,45	0,00	31	500	100,00	0,00
CP003068.1	*S. pyogenes* Alab49	97,44	0,00	968155	967687	99,36	0,00

* SDSD VSD6 ***speL*** sequence (GenBank accession HQ724303.1) was used as reference for alignment

Table S7. Nucleotide sequence identity (%) obtained from multiple alignment of ***speM*** sequences the sequences of the streptococcal species deposited in the GenBank database using BlastN.

ID	DESCRIPTION	%IDENTITY	E-VALUE	BIT SCORE	HSP START	HSP END	OVERLAP
HQ724304.1	SDSD pyrogenic exotoxin M (speM)*	100,00	0,00	953,47	1	528	100,00
HQ724305.1	SDSD pyrogenic exotoxin M (speM)	99,81	0,00	948,96	1	528	100,00
AB074529.1	SDSD sdm gene for mitogen	99,43	0,00	939,94	217	744	100,00
LR134094.1	SDSD strain NCTC4669	98,86	0,00	924,61	1863313	1862789	99,05
CP035455.1	*S. pyogenes* strain emm197	98,11	0,00	906,58	847420	847944	99,43
CP035454.1	*S. pyogenes* strain emm54	98,11	0,00	906,58	968369	967845	99,05
LS483414.1	*S. pyogenes* strain NCTC13736	98,11	0,00	906,58	901545	902069	99,43
LS483338.1	*S. pyogenes* strain NCTC12064	98,11	0,00	906,58	791111	791635	99,43
CP010449.1	*S. pyogenes* strain NGAS322	98,11	0,00	906,58	1009544	1009020	99,05
CP007240.1	*S. pyogenes* strain 7F7	98,11	0,00	906,58	941898	941374	99,05
CP007023.1	*S. pyogenes* STAB1102 genome	98,11	0,00	906,58	941624	941100	99,05
CP006366.1	*S. pyogenes* HSC5	98,11	0,00	906,58	966153	965629	99,05
CP003068.1	*S. pyogenes* Alab49	98,11	0,00	906,58	969139	968615	99,05
CP047120.1	*S. pyogenes* strain 1085	97,92	0,00	902,07	465466	465990	99,43
CP031628.1	*S. pyogenes* strain MGAS28330	97,92	0,00	902,07	845163	845687	99,43
CP033335.1	*S. pyogenes* strain TSPY208	97,92	0,00	902,07	983143	982619	99,05
CP033336.1	*S. pyogenes* strain TSPY165	97,92	0,00	902,07	684744	685268	99,43
LR134272.1	*S. pyogenes* strain NCTC12060	97,92	0,00	902,07	953610	953086	99,05
LS483391.1	*S. pyogenes* strain NCTC8320	97,92	0,00	902,07	1009172	1008648	99,05
LS483389.1	*S. pyogenes* strain NCTC10879	97,92	0,00	902,07	823946	824470	99,43
LS483356.1	*S. pyogenes* strain NCTC8230	97,92	0,00	902,07	1054475	1053951	99,05
LS483352.1	*S. pyogenes* strain NCTC12052	97,92	0,00	902,07	1020984	1020460	99,05
CP021640.1	*S. pyogenes* strain JS12	97,92	0,00	902,07	976115	975591	99,05
AE009949.1	*S. pyogenes* MGAS8232	97,92	0,00	902,07	1043252	1042728	99,05
CP033621.1	*S. pyogenes* strain M75	97,73	0,00	897,56	1146492	1145968	99,05
LS483437.1	*S. pyogenes* strain NCTC13751	97,73	0,00	897,56	1185059	1184535	99,05

*SDSD VSD7 ***speM*** sequence (GenBank accession HQ724304.1) was used as reference for alignment
